# RpoS-regulated *SEN1538* gene promotes resistance to stress and influences *Salmonella enterica* serovar enteritidis virulence

**DOI:** 10.1080/21505594.2020.1743540

**Published:** 2020-04-05

**Authors:** Aryashree Arunima, Sunil Kumar Swain, Shilpa Ray, Birendra Kumar Prusty, Mrutyunjay Suar

**Affiliations:** a School of Biotechnology, Kalinga Institute of Industrial Technology, Bhubaneswar, India; b District Diagnostic Laboratory, Malkangiri, Odisha, India

**Keywords:** *Salmonella* Enteritidis, SEN1538, heat stress, antimicrobial peptide stress, SPI-1, SPI-2, C57BL/6, virulence, inflammation, Illumina Hiseq4000

## Abstract

*Salmonella enterica* serovar Enteritidis (*S*. Enteritidis; wild type (WT)) is a major cause of foodborne illness globally. The ability of this pathogen to survive stress inside and outside the host, such as encountering antimicrobial peptides and heat stress, determines the efficiency of enteric infection. These stressors concertedly trigger virulence factors encoded on *Salmonella* pathogenicity islands (SPIs). Although RpoS is a well-known central transcriptional stress and virulence regulator, functional information regarding the genes of the regulon is currently limited. Here, we identified *SEN1538* as a conserved RpoS-regulated gene belonging to the KGG protein superfamily. We further assessed its role in pathogenic stress responses and virulence. When *SEN1538* was deleted (Δ1538), the pathogen showed reduced survival during antimicrobial peptide introduction and heat stress at 55°C compared to WT. The mutant displayed 70% reduced invasion in the HCT116 colon epithelial cell line, 5-fold attenuated phagocytic survival in RAW264.7 cells, and downregulation of several SPI-1 and SPI-2 genes encoding the three secretion system apparatus and effector proteins. Δ1538 also showed decreased virulence compared to WT, demonstrated by its reduced bacterial counts in the feces, mLN, spleen, and cecum of C57BL/6 mice. Comparative transcriptomic analysis of Δ1538 against WT revealed 111 differentially regulated genes, 103 of which were downregulated (fold change ≤ −1.5, P < 0.05). The majority of these genes were in clusters for metabolism, transporters, and pathogenesis, driving pathogenic stress responses and virulence. *SEN1538* is, therefore, an important virulence determinant contributing to the resilience of *S*. Enteritidis to stress factors during infection.

## Introduction

During their life cycle, *Salmonella enterica* subspecies I serovars (*Salmonella*) experience a wide range of stress factors in the environment and inside the host. This entails changes in temperature (encountered in food and inside hosts), action of antimicrobial peptides (AMPs) (in the oral cavity, liver, small intestine, and phagocytes), low pH, bile salts, nutrient scarcity, reactive oxygen species, and nitrosative stress[1]. After ingestion by the host, *Salmonella* survives in these hostile conditions, which determine its virulence and the efficiency of enteric infection[1]. The adaptive mechanisms by which pathogens respond to stress require intricate cross talk between bacterial stress response pathways and the virulence factors spanning across the *Salmonella* pathogenicity islands (SPIs) [1,2]. Although *Salmonella* pathogenesis has been extensively studied in *Salmonella enterica* subspecies I serovar Typhimurium (*S*. Typhimurium), they have not been studied in *Salmonella enterica* subspecies I serovar Enteritidis (*S*. Enteritidis) [3]. Of the two serovars, *S*. Enteritidis has emerged as the more prevalent serovar and is a major contaminant in the poultry and meat industry [4,5].

Low pH, AMPs, low intracellular Mg^2+^, bile salts, and oxidative and nitrosative stress are potent activators of sensor kinase systems, which trigger the expression of virulence genes encoded on SPI-1 and SPI-2 [1,2,6]. SPI-1 is required for pathogenic invasion into host intestinal epithelia, while SPI-2 facilitates intra-macrophage survival and systemic dissemination in the host [3]. Major deviations in temperature from the optimal growth temperature (37°C) are also an important environmental stress signal for foodborne pathogens like *Salmonella* [1]. *S*. Enteritidis are killed at a temperature ≥70°C [7], but can resist and survive high temperatures up to 55°C [8,9]. The pathogen can survive temperature fluctuations by activating a set of stress response genes [10,11]. This response to heat stress also triggers virulence gene expression. Taken together, these factors determine the ability of the pathogen to survive under harsh conditions [7,9,10,12].

RpoS is a central transcriptional regulator for stress, and is conserved across several Gram-negative pathogens. It controls the hierarchical expression of different stress response pathways and virulence factors in pathogens, allowing for their survival and pathogenesis [11,13]. Several studies in *S*. Typhimurium and *Escherichia coli* (*E. coli*) have revealed a vast set of genes regulated under RpoS with unknown functions [14]. The three paralogous small proteins YciG, YmdF, and STM1513/STM14_1829 were identified in several regulatory studies on RpoS [14–16]. These three proteins belong to the KGG protein superfamily, and have been predicted to play a role in the general stress response and flagellum-dependent motility of *S*. Typhimurium [13,14,16–19]. YciG was previously reported to confer resistance to heat and acid stress in *S*. Typhimurium and *E. coli* [17,20,21]. Furthermore, STM14_1829 was reported to play a role in the swimming motility of S. Typhimurium ATCC14028 [19]. Nonetheless, the role of the KGG protein family in bacterial stress pathways and pathogenesis still remains largely unexplored, especially in *S*. Enteritidis.

In the present study, we identified orthologs of YciG, YmdF, and STM1513/STM14_1829 in *S*. Enteritidis through BLASTp and synteny analysis. SEN1538 protein was identified as an ortholog of STM1513/STM14_1829 in *S*. Enteritidis. Interestingly, SEN1538 and its paralogs did not play any role in the flagellum-dependent motility of *S*. Enteritidis. We selected SEN1538 to further investigate its role in the stress response and virulence of the pathogen. This was achieved through gene deletion and complementation analysis of *SEN1538 (*Δ1538) under heat stress, AMP stress, and other intracellular stress conditions. Further, the virulence of Δ1538 was assessed in a streptomycin pre-treated C57BL/6 mouse infection model [22]. Comparative global transcriptome analysis was also conducted in Δ1538 to gain insight into the role of SEN1538 in bacterial adaptation to stress. Transcriptomic profiling revealed a subset of differentially expressed genes involved in metabolism, transport, and pathogenesis. In conclusion, we determined that SEN1538 was important for bacterial survival during stress, invasion into host epithelia, replication inside macrophages, and systemic infection in a murine typhoid model.

## Materials and methods

### Bioinformatic analysis

The SEN1538 protein (GenBank Accession ID: CAR33117.1, RefSeq Accession ID: WP_000807638.1) of *Salmonella enterica* subspecies I serovar Enteritidis str. P125109 was identified as an ortholog of STM1513/STM14_1829 through BLASTp analysis at NCBI (National Centre for Biotechnology Information; http://www.ncbi.nlm.nih.gov), using the STM1513/STM14_1829 protein sequence (GenBank Accession ID: AAL20432.1) from *Salmonella enterica* subspecies I serovar Typhimurium str. LT2 as the query. Pairwise sequence alignment between the two proteins was done using MAFFT v. 7 (http://mafft.cbrc.jp/alignment/server/). Synteny analysis was conducted using standard gene visualization tools at NCBI. Domain and motif conservation were validated through the analysis of sequences in the Pfam (http://pfam.sanger.ac.uk/) and InterProScan5 databases. During BLAST analysis against the NCBI non-redundant database (E value 0.01), homologous sequences to CAR33117.1 were found using *Salmonella enterica* subspecies I serovar Enteritidis str. P125109 as the query. Multiple alignment analysis was performed using MAFFT v. 7 (http://mafft.cbrc.jp/alignment/server/). The alignments obtained were manually adjusted by BioEdit software v.7.1. The final optimized multiple sequence alignment output was then visualized using Jalview (http://www.jalview.org) software.

### Bacterial strains and growth conditions

Bacterial strains (Table 1) were grown in an incubator (New Brunswick™ Innova® 42 R Incubator and Refrigerated Shaker, Eppendorf^TM^ #M1335-0016) under different culture conditions for subsequent experiments. M9 minimal medium (20% 5X M9 salt (211.3 mM Na_2_ HPO_4_, 110 mM KH_2_PO_4_, 42.77 mM NaCl, 93.4 mM NH_4_ Cl), 2 mM MgSO_4_, 0.1 mM CaCl_2_, 0.4% glucose, 0.1% casamino acid) was used for bacterial stress response assays [20]. For the *in vitro* invasion assay and studying SPI-1 gene expression, bacterial strains were grown in Luria-Bertani (LB) medium containing 0.3 M NaCl at 37°C under static conditions (no shaking) for 12 h (SPI-1 inducing medium) [23]. Strains were subcultured in fresh SPI-1 inducing medium at a 1:20 ratio until an optical density (O D_600_) of 0.6 was attained. Macrophage survival, an intra-macrophage survival assay, and study of SPI-2 gene expression was carried out by culturing bacterial strains in SPI-2 inducing media (5 mM KCl, 7.5 mM (NH_4_)_2_SO_4_, 80 mM MES, 38 mM glycerol, 0.1% casamino acids, 24 mM MgCl_2_, 337 mM PO4^3-^) [24] at 37°C and 150 rpm for 12 h.

**Table 1.** Strains and plasmids used in the study.

Strains or Plasmids	Relevant genotype and/or phenotype	Background	Resistance	References
**Strains**				
WT	*Salmonella enterica* subspecies I serovar Enteritidis str. P125109 (*S*. Enteritidis)	Wild type	Sm^r^ (naturally resistant)	[31]
ΔinvC	TTSS-1^−^ (*invC:: aphT*)	Wild type	Sm^r^ Km^r^	[31]
ΔssaV	TTSS-2^−^ (*ssaV::aphT*)	Wild type	Sm^r^ Km^r^	[23]
Δ1538	*SEN1538* mutant (*SEN1538::aphT*)	Wild type	Sm^r^ Km^r^	This study
ΔrpoS	*rpoS* mutant (*rpoS:: aphT*)	Wild type	Sm^r^ Km^r^	This study
cΔ1538	pCH112_1538 transformed into Δ1538 to rescue the function of *SEN1538*	Δ1538	Sm^r^ Km^r^ Amp^r^	This study
WT-SEN1538His	*SEN1538* chromosomally his-tagged at C-terminal end	Wild type	Sm^r^ Km^r^	This study
ΔyciG	*yciG* mutant (yciG::*aphT*)	Wild type	Sm^r^ Km^r^	This study
ΔymdF	*ymdF* mutant (*ymdF:: aphT*)	Wild type	Sm^r^ Km^r^	This study
SB300:ΔyciG	*yciG* mutant (*yciG::cat*)	SB300	Sm^r^Cm^r^	[20]
SB300:ΔflgD	*flgD* mutant (*flgD::cat*)	SB300	Sm^r^Cm^r^	[20]
**Plasmids**				
pKD4	*bla* FRT *aphT* FRT PS1 PS2 *ori_R6_ _K_*		Km^r^	[25]
pKD46	*bla* P_BAD_ *gam bet exo* pSC101 *ori_TS_*		Amp^r^	[25]
pCJLA	GFP tag expressing plasmid		Km^r^ Cm^r^	[31]
pCH112	*hilA* ORF cloned into P_BAD_/*myc*-His; *ori_pBR322_*	pCH112	Amp^r^	[23]
pCH112_1538	*SEN1538* ORF with 1000-bp upstream region cloned into pCH112 between NcoI and XbaI by replacing the ORF of *hilA; ori_pBR322_*	pCH112	Amp^r^	This Study

**Sm^r^: Streptomycin resistance; Km^r^: Kanamycin resistance; Amp^r^: Ampicillin resistance; Cm^r^: Chloramphenicol resistance**

For growth curve analysis, overnight cultures of bacterial strains were grown in LB and M9 minimal medium at 37°C and 150 rpm. Strains were subcultured at a ratio of 1:100. Bacterial counts and the OD. were obtained at hourly intervals from 0 to 10 h. Bacterial counts were obtained through serial dilution and plating on LB agar. Data were represented in cfu/mL. The bacterial OD. was obtained at 600 nM using an Agilent®Cary 60 UV-Vis Spectrophotometer. Experiments were performed in triplicate.

### Construction of isogenic mutant- and plasmid-based complementation

Chromosomal deletion of the genes *SEN1538, yciG, ymdF*, and *rpoS* from the *Salmonella enterica* subspecies I serovar Enteritidis str. P125109 (*S*. Enteritidis; WT) genome was achieved using λ-red recombinase mutagenesis, with pKD4 (*aphT*; Kanamycin resistance cassette) as the template plasmid [25]. Primers used for construction and confirmation of isogenic deletion and nonpolar mutant Δ1538, ΔyciG, ΔymdF, and ΔrpoS are listed in Supplementary Table 1 (Table S1). The mutants were confirmed through polymerase chain reaction (PCR) using an internal primer annealing to the kanamycin cassette and a forward confirmatory primer (Table S1) annealing 200 bp upstream of the target gene. Mutants were confirmed by generating a final 1200 bp PCR product.

For complementation of the deletion strain Δ1538, plasmid pCH112_SEN1538 was constructed by manipulating the pCH112 vector [23]. Briefly, the *SEN1538* gene (size: 183 bp), along with its 1000 bp upstream sequence (native promoter), was amplified via PCR using primers listed in Table S1. The resulting amplicon and pCH112 vector were digested with restriction enzymes NcoI and XbaI. The digested insert was cloned into the pCH112 vector, replacing the open reading frame (ORF) of *hilA* with the ORF of *SEN1538* and its promoter sequence. The resultant cloned construct pCH112_1538 was confirmed by observing a 1183 bp insert fallout. The pCH112_1538 plasmid was transformed into deletion strain Δ1538 to rescue the function of *SEN1538*, resulting in the complementation strain cΔ1538.

### *6X-His tagging of chromosomal* SEN1538 *gene*

Recombinational transfer of the 6X-His tag sequence into WT was achieved by using a modified protocol of λ-red recombinase mutagenesis [25,26]. The forward primer for chromosomal tagging comprises 40–45 nucleotide extensions at the 5ʹ end, which is homologous to the last portion of *SEN1538* (herein termed as F1 primer). The nucleotide sequence coding for the 6X-His tag was added at the 3ʹ end of the F1 primer and before the stop codon in the F1 primer, which is herein named the F2 primer (5ʹ to 3ʹ direction; 42 nucleotide extensions homologous to the last portion of the target gene + 6X-His tag nucleotide sequence + stop codon of *SEN1538*). The FRT sequence was added to the 3ʹ end of the F2 primer to generate the final forward primer “FwSEN1538-His” (Table S1). For the reverse primer, a 45-nucleotide bp homologous to the region immediately downstream of the *SEN1538* stop codon was designed (herein termed R1 primer). The FRT sequence was added to the 3ʹ end of the R1 primer and was reverse complemented to generate reverse primer “RwSEN1538-His”. The FwSEN1538-His and RwSEN1538-His primers were used for amplifying the kanamycin resistance cassette from the pKD4 plasmid using the PCR conditions described by Datsenko and Wanner [25]. The final amplicon comprised of a kanamycin resistance cassette with a 5ʹ overhang (42 nucleotide extensions homologous to the last portion of the target gene + 6X-His tag nucleotide sequence + stop codon) and 3ʹ overhang (45 nucleotide extensions homologous to the downstream region immediate to the stop codon of the target gene). The 1500 bp amplicon was precipitated with 100% molecular grade ethanol (EMSURE, MERCK). The precipitated product was resuspended in nuclease-free water (Qiagen) and transformed into the electrocompetent cells of WT, which was harboring the helper plasmid pKD46. Following transformation, these cultures were grown at 30°C and 150 rpm for 3 h. The transformants were selected by plating all bacterial cells on the LB plates supplemented with kanamycin (50 µg/mL). His tagging of *SEN1538* in WT (WT-SEN1538His; Table 1) was confirmed through PCR using a confirmatory forward primer (Confo SEN1538-His) annealing to the 6X-His tag sequence of the *SEN1538* and reverse primer (RwSEN1538-His). This generated a final amplicon of size ~1500 bp. The primer sequences and strain information used for chromosomal His-tagged strain construction are listed in Table S1 and Table 1. An illustration of the method for generating chromosomal 6X-His tagging of *SEN1538* is provided in Supplementary Figure 1 (Figure S1).

### Stress survival assay

#### Antimicrobial peptide sensitivity assay

The AMP sensitivity assays were performed as previously described [27]. Briefly, overnight cultures of WT, Δ1538, and the complemented strain cΔ1538 were subcultured (1:100) in LB medium at 37°C and 150 rpm until 0.4 OD._600_ (log phase). Strains were then diluted with 1X phosphate-buffered saline (PBS) to obtain 10^8^ cfu/mL. Each strain suspension was then incubated at 37°C for 1 h with 1 µg/mL AMP, 1 µg/mL polymyxin B (Himedia, India), and 10 µg/mL LL-37 (Sigma). Counts for the number of surviving bacteria were obtained by serial dilutions and plating on LB agar. The bacterial survival percentage was obtained by dividing the colony-forming units (CFU) post-AMP treatment against the CFU pre-AMP treatment, with WT survival normalized to 100%.

#### Heat stress assay

A heat stress assay was performed as previously described [8,9]. Overnight cultures of WT, Δ1538, and the complemented strain cΔ1538 were grown in M9 minimal medium at 37°C and 150 rpm. Strains were subcultured in M9 minimal medium at 37°C and 150 rpm until log phase. These cultures were subjected to a high temperature of 55°C and 150 rpm for up to 4 h. Surviving bacterial counts following heat stress were enumerated through serial dilutions and plating on LB agar for up to 4 h. The bacterial survival percentage was obtained by comparing the CFU of Δ1538 and the complemented strain cΔ1538 against WT (normalized to 100%) at indicated time points.

#### Magnesium ion starvation assay

The log-phase cultures of WT, Δ1538, and the complemented strain cΔ1538 were transferred into fresh M9 minimal medium with a low concentration of MgSO_4_ (20 µM). The strains were also grown in an optimal concentration of MgSO_4_ (200 µM), which served as the experimental control. Surviving bacterial counts following magnesium starvation were enumerated through serial dilutions and plating on LB agar for up to 4 h. The bacterial survival percentage was obtained by comparing the CFU of Δ1538 and the complemented strain cΔ1538 against WT (normalized to 100%) at indicated time points.

#### Bile stress assay

Bile stress assays were performed as previously described [28]. Log-phase cultures of WT, Δ1538, and the complemented strain cΔ1538 grown in M9 minimal medium were diluted to obtain 10^8^ cfu/mL. These strains were incubated at 37°C for up to 4 h with bile salt mixture (15%; Himedia, India). Surviving bacterial counts after subjecting to bile stress were enumerated through serial dilutions and plating on LB agar for up to 4 h. The bacterial survival percentage was obtained by comparing the CFU of Δ1538 and the complemented strain cΔ1538 against WT (normalized to 100%) at indicated time points.

#### Acid stress assay

Overnight cultures of WT, Δ1538, and the complemented strain cΔ1538 were subcultured in glucose MEM (pH 7.4) at 37°C and 150 rpm until log phase. These cultures were challenged with pH 3 (± 0.1) and pH 5 (± 0.2) with 3 N HCl. Surviving bacterial counts post-treatment with acid stress were enumerated through serial dilutions and plating on LB agar for up to 4 h. The bacterial survival percentage was obtained by comparing the CFU of Δ1538 and the complemented strain cΔ1538 against WT (normalized to 100%) at indicated time points.

### Western blot analysis

To study the role of the protein SEN1538 during heat stress, log-phase cultures of WT-SEN1538His were subjected to a high temperature of 55°C for 2 h and then compared to the strains grown at 37°C and 150 rpm. To study SEN1538 expression during AMP stress, a log-phase culture of WT-SEN1538His was treated with PMB (1 µg/mL) for 1 h and compared to untreated culture. Whole cell bacterial lysates from the stressed and control cultures of WT-SEN1538His were obtained. These bacterial lysates were subjected to electrophoretic separation on a 12% SDS polyacrylamide gel. The protein on the gel was transferred to poly(vinylidene difluoride) membrane (GE Healthcare & Life Sciences) and probed with polyclonal anti-His antibody [1:6000 (Abcam)] and polyclonal anti-RpoA antibody (1: 15,000), followed by probing with secondary antibodies. Detection was performed using enhanced chemiluminiscence (ECL western blotting substrate kit (Abcam)). The expected molecular mass of the 6X-His-tag fusion protein of SEN1538 is 9 KDa, and the molecular mass of RpoA is 37 KDa. RpoA served as the internal loading control for the experiments.

### Adhesion and invasion assay

Adhesion and invasion assays were performed as previously described [20]. Briefly, the HCT116 colon epithelial cell line was cultured in Dulbecco’s modified Eagle medium (DMEM) (Gibco, Germany) supplemented with 10% fetal bovine serum (FBS, Gibco) and antibiotic (Antibiotic solution 100X liquid, Himedia® #A001A) at 37°C and 5% CO_2_. Cells were seeded in 24-well plates and grown until a confluence of 80% was obtained. Prior to infection, culture medium was removed and cells were washed twice with DMEM, followed by addition of DMEM without antibiotics. WT, Δ1538, and the complemented strain cΔ1538 were grown in SPI-1 inducing medium for infection until an OD_600_ of 0.6 was reached. Bacterial counts were obtained by serial dilutions and plating on LB agar.

Adhesion assays were performed by incubating both the bacterial inoculums and HCT116 cells on ice for 30 min prior to infection. HCT116 cells were subsequently infected at a multiplicity of infection (MOI) of 10, followed by incubation on ice for 30 min. HCT116 cells were immediately lysed after 30 min of infection with 0.1% sodium deoxycholate diluted in PBS, and appropriate dilutions were plated on LB agar. For the invasion assay, HCT116 cells were infected at a MOI of 10, followed by incubation for 50 min at 37°C and 5% CO_2_ in culture medium without DMEM. Cells were washed twice with medium followed by addition of DMEM-containing gentamicin (100 µg/mL) and incubation for 2 h. Post-gentamicin protection, cells were washed twice with 1X PBS and lysed in 0.1% sodium desoxycholate in PBS, and appropriate dilutions were plated on LB agar. The percentage of invasion and adhesion was obtained by dividing the CFU recovered by the number of bacteria infected, followed by multiplying the result by 100. Adhesion (%) and invasion (%) for Δ1538 and the complemented strain cΔ1538 was compared to WT (normalized to 100%).

### Macrophage uptake and intra-macrophage survival assay

Macrophage uptake and replication assays were performed as previously described [20]. Briefly, RAW264.7 murine macrophage cells were seeded in 24-well plates and cultured in DMEM supplemented with 10% FBS without antibiotics at 37°C and 5% CO_2_. This was done until 80% confluence was achieved. WT, Δ1538, and complemented strain cΔ1538 were cultured in SPI-2 inducing medium for infection, and counts were obtained by serial dilutions and plating on LB agar. Subsequently, RAW264.7 cells were infected at a MOI of 10 and incubated for 50 min. Cells were washed twice with medium without antibiotics, followed by addition of DMEM-containing gentamicin (100 µg/mL) and incubation for 2 h. Bacterial uptake was calculated by lysing RAW264.7 cells with 0.1% Triton-X 100 in PBS after 2 h of gentamicin treatment. Serial dilutions were made and plated on LB agar. For the intra-macrophage survival assay, after 2 h of incubation, RAW264.7 cells were washed with medium without antibiotics and were incubated in DMEM containing gentamicin (10 µg/mL) for 24 h. The RAW264.7 cells were lysed, and serial dilutions were plated to enumerate the number of surviving bacteria. The percentage of uptake was obtained by dividing the CFU recovered at 2 h by the number of infected bacteria, followed by multiplying the result by 100. The bacterial uptake for Δ1538 and the complemented strain cΔ1538 was compared to WT (uptake normalized to 100%). The intracellular survival of bacterial strains was calculated by dividing the CFU recovered at 24 h by CFU recovered post-uptake. The bacterial survival of Δ1538 and complemented strain cΔ1538 was represented as fold replication in comparison to WT survival at 24 h.

### Flow cytometry

WT, Δ1538, and the complemented strain cΔ1538 were transformed with green fluorescent protein (GFP)-expressing plasmid pCJLA [29,30], which generated WT-pCJLA, Δ1538-pCJLA, and the complemented strain cΔ1538-pCJLA (Table 1). For the invasion assay, HCT116 cells were infected with WT-pCJLA, Δ1538-pCJLA, and the complemented strain cΔ1538-pCJLA at a MOI of 50 [29,31] for 50 min in DMEM without antibiotics. The medium was removed after 50 min, and the cells were incubated in DMEM containing gentamicin (100 µg/mL) for 2 h. Post-gentamicin treatment, HCT116 cells were washed with medium without antibiotics. The intensity of GFP was monitored and the mean fluorescence intensity (MFI) of GFP expression was plotted. For macrophage uptake and replication assay, RAW264.7 cells were infected with WT-pCJLA, Δ1538-pCJLA, and the complemented strain cΔ1538-pCJLA at a MOI of 50 [29,31] for 50 min. DMEM was removed after 50 min, and the RAW264.7 cells were incubated in DMEM containing gentamicin (100 µg/mL) for 2 h. For cellular uptake, cells were washed after 2 h with medium without antibiotics. The number of internalized bacteria was monitored via the MFI of GFP expression in the infected RAW264.7 cell line. Similarly, for the intra-macrophage survival assay, after treatment with gentamicin (100 µg/mL) for 2 h, cells were incubated in medium containing gentamicin (10 µg/mL) for 24 h. After 24 h, RAW264.7 cells were recovered and survival was monitored. Cell lines not infected with GFP-expressing bacterial strains were taken as the uninfected and non-fluorescent control for the experiments (Figure S2). The gating strategy for all flow cytometric assays is depicted in Figure S2. Invasion, bacterial uptake, and survival data were obtained in terms of MFI (%) and were compared to cells infected with WT-pCJLA. Data were acquired using BD FACScanto™ II cytometer (Becton–Dickinson, Erembodegem, Belgium) and analyzed using Flowjo v. 10.4.2.

### Motility assay

To conduct bacterial swimming motility assays, strains were grown overnight and CFU of strains were adjusted to 10^8^ cfu/mL. We spotted 1.5 μl of the bacterial cultures on soft LB agar plates (0.3% w/v) LB agar) and incubated them at 37°C for 5 h [29]. The diameters of motile cell growth were measured, and the experiment was repeated in triplicate.

### Ethical approval

All mice were maintained, and infection experiments were performed, in strict accordance with the guidelines of the Institutional Animal Ethics Committee (IAEC), Kalinga Institute of Industrial Technology (KIIT), under the license number KSBT/IAEC/2017/MEET-2/A1.

### C57BL/6 mice infection experiment

C57BL/6 specific pathogen-free (SPF) mice were housed in a ventilated cage facility at the School of Biotechnology, KIIT. Mice aged 6–8 weeks were pre-treated with streptomycin intragastrically at a dosage of 50 mg, as previously described [22,23]. WT, Δ1538, and the complemented strain cΔ1538 were grown overnight in LB and subcultured (1:20) until an OD._600_ of 0.6 was achieved. The bacterial inoculums (~10^7^ CFU) from these strains were prepared for orogastric inoculation of each mice group (n = 5). Fecal bacterial load was determined at 24 and 48 h post-infection (p.i.) by serial dilutions and plating on MacConkey agar plates. Fecal samples were collected at 72 h p.i. from all mice groups for lipocalin-2 estimation, and mice were euthanized for organ collection. Bacterial load in the cecum, mesenteric lymph node (mLN), spleen, and liver was determined by plating appropriate dilutions of the organ homogenates on MacConkey agar with required antibiotics. Tissue segments of the ileum, cecum, and colon were fixed and embedded in an optimum cutting temperature (OCT) (Sakura Finetek Inc.) by snap freezing in liquid nitrogen, and were stored at −80°C for cryosectioning.

### Histopathological evaluation

The cryofixed and embedded cecal segments were sectioned (size; 5 µM) at −30°C. The sections were placed on glass slides and allowed to dry for at least 2 h at 25°C before staining with hematoxylin and eosin (H&E). The stained histopathological sections were evaluated based on a previously described scoring system for analysis of cecal inflammation [23,31]. Briefly, H&E-stained sections (5 µM) were independently assessed and scored based on parameters such as submucosal edema, polymorphonuclear neutrophil (PMN) infiltration, loss of goblet cells, and epithelial ulceration. The pathoscore of individual mice from each group (n = 5) was evaluated and averaged to assign an independent pathoscore to each group. Pathological scores ranged from 0 to 13 and denoted varying degrees of inflammation as per the following: intact and non-inflamed intestine (0); minimal inflammation (1–2); slight inflammation (3–4); moderate inflammation (5–8); and significant inflammation (9–13) [23]. H&E images were acquired using ZEISS Apotome.2 with a scale bar of 200 µM.

### Lipocalin-2 estimation

Lipocalin-2 was measured via an enzyme-linked immunosorbent assay (ELISA) from a 72-h p.i. fecal sample using a Thermo Scientific™ Pierce™ Mouse Lipocalin-2 (LCN2) ELISA Kit. Briefly, fecal samples collected at 72 h p.i. were homogenized in 500 µL PBS. These samples were centrifuged at 7,000 rpm for 15 min (Eppendorf, 5424 R), and the fecal supernatants were collected and stored at −80°C for storage. Fecal supernatants and standards were diluted and the experiment was performed according to the manufacturer’s instructions. The final absorbance was acquired using a Thermo Scientific™ Multiskan™ GO Microplate Spectrophotometer at λ_max_ of 450 nM and 550 nM. The concentration of lipocalin-2 was obtained by interpolating the values from the standard plot. Lipocalin-2 values are expressed as log ng per gram of feces.

### Serum cytokine analysis

A serum cytokine assay was performed using the blood serum of mice. Blood was drawn from mice and serum was collected at 72 h p.i. A cytokine assay was performed using a MILLIPLEX MAP Mouse Cytokine/Chemokine Magnetic Bead Panel – Premixed 32 Plex Immunology Multiplex Assay. The experiment was performed according to the manufacturer’s instructions, and data were acquired using a Biorad Bioplex 200 system.

### Isolation of bacterial RNA

To study the expression of *SEN1538* and *rpoS* under different stress conditions, log-phase cultures of WT were treated with AMP stress, heat stress, magnesium starvation stress, and bile stress. The expression of the genes during stress was compared to the WT grown under normal conditions (control; WT grown at 37°C and 150 rpm in LB or M9 minimal medium). After 30 min of stress treatment, the treated cultures and control were centrifuged at 13,000 rpm for 1 min (Eppendorf, 5424 R) to obtain a bacterial pellet.

To study the effect of *SEN1538* deletion on stress response genes, a log-phase culture of Δ1538 was treated with heat and AMP stress and compared to WT. After 30 min of exposure to these stresses, cultures were centrifuged at 13,000 rpm for 1 min to obtain the bacterial pellet. RNA was extracted from the bacterial pellets using QIAzol Lysis Reagent (Qiagen, Germany) and resuspended in nuclease-free water (Qiagen, Germany). To remove contamination of bacterial DNA, extracted RNA was treated with RNAse-free DNAse I (Thermo Scientific™, #EN0521).

To study the role of RpoS in *SEN1538* regulation, log-phase cultures of WT and ΔrpoS were subjected to different stress conditions. After 30 min of exposure to stress, bacterial cultures were centrifuged at 13,000 rpm for 1 min to obtain the bacterial pellet. RNA was isolated from the pellet of WT and ΔrpoS using QIAzol Lysis Reagent (Qiagen, Germany) followed by RNase-free DNase I treatment (Thermo Scientific™, #EN0521) to obtain pure RNA.

To check the effect on expression of SPI-1 and SPI-2 genes in Δ1538, the mutant and WT were grown in SPI-inducing conditions, and RNA was isolated for real-time PCR analysis. To check the expression of *SEN1538* in WT during infection, HCT116 and RAW264.7 cell lines were infected with WT at a MOI of 50. DMEM was removed after infection at indicated time points (see Figures 3 and 4). HCT116 and RAW264.7 cells were washed with 1X PBS to remove non-internalized bacteria. The cells were recovered and lysed with QIAzol Lysis Reagent for RNA, resuspended in nuclease-free water (Qiagen, Germany), and treated with RNAse-free DNAse I for pure RNA.

### qRT-PCR analysis

RNA was quantified using a Colibri® microvolume spectrometer (TITERTEK BERTHOLD) and normalized for cDNA synthesis. cDNA synthesis was performed using a Verso cDNA Synthesis Kit (Thermo Scientific™, Cat# AB-1453/A). Quantitative real-time PCR (qRT-PCR) was carried out using synthesized cDNA as a template and a 2X DyNAmo ColorFlash SYBR Green qPCR Kit (Thermo Scientific™) in Realplex4 epgradient Mastercycler (Eppendorf). Primers used for qRT-PCR analysis are listed in Table S1. Relative quantification of gene expression was done via the ^ΔΔ^Ct method. The *16 s rRNA* gene was used as the housekeeping gene in our study. Log_2_ fold change cutoff ≥1.5 was considered to indicate upregulation, and ≤ −1.5 was considered to indicate downregulation.

### Library preparation and sequencing

For global transcriptome analysis of WT and Δ1538, an overnight culture of these strains was subcultured at 37°C and 150 rpm in LB until log phase. These cultures were subsequently snap-frozen in liquid nitrogen. RNA extraction was carried out from the snap-frozen cultures of WT and Δ1538 using QIAzol Lysis Reagent. Extracted RNA was resuspended in nuclease-free water (Qiagen), and DNA contamination was removed by RNase-free DNAse I (Thermo Scientific™, #EN0521). A RNA integrity number greater than 6 was used for further workflow.

RNA-seq library preparation from RNA samples of WT and Δ1538 was carried out with biological duplicates using the Illumina recommended protocol. The cDNA library was generated and sequenced using a 101 bp paired-end sequencing module in the Illumina HiSeq4000 library sequencing platform at Bionivid Technology Pvt. Ltd. (India). Following sequencing, quality control of the raw data was performed using the NGS QC Toolkit [32] to ensure that at least 70% of the total bases per read had a Phred quality score of ≥30.

### Mapping of reads and alignment to reference genome for transcriptomic analysis

Bacterial transcriptomic analysis was performed by aligning the high quality filtered reads obtained from sample duplicates of WT and Δ1538. A total of 259.45 million paired-end reads were obtained, of which WT had an average of 55.64 million high-quality sequence reads, and Δ1538 had an average of 52.3 million. The paired-end high-quality sequence reads of WT and Δ1538 were mapped to a reference genome of *Salmonella enterica* subspecies I serovar Enteritidis str. P125109 (Accession No. AM933172.1) using the Rockhopper62 tool [33]. Rockhopper reference-based assembly provides information about the reads aligning to the reference genome and assembling transcripts. It identifies assembling transcripts and novel transcripts such as micro RNA and small RNA.

An average of 97.5% of the high quality reads from sample duplicates of Δ1538, and 98% of WT high quality reads, aligned well with the reference genome. The RNA-seq data obtained from sample duplicates of Δ1538 were compared with the sample duplicates of WT. Data from each sample were normalized by the total read count and reads per kilobase per million mapped sequence reads (RPKM) values. Genes were considered to be differentially expressed on the basis of RPKM > 1 in either of the pair of samples, with Log_2_ fold change cutoff set to ≥1.5 (for upregulation) or ≤ −1.5 (for downregulation). The fold change was considered statistically significant at P < 0.05.

### Gene Ontology (GO) annotation analysis of transcriptome data

Gene ontology (GO) annotation for genes in the RNA-seq study was performed using UniProt (http://www.uniprot.org/). Differentially expressed genes obtained from RNA-seq analysis were clustered using the Top 5 GO and Kyoto Encyclopedia of Genes and Genomes (KGG) databases (https://www.genome.jp/kegg/pathway.html) based on their molecular functions, biological processes, cellular components, and pathways.

### Statistical analysis

All experiments were performed in triplicate unless otherwise stated. Data are presented as the mean ± standard deviation (SD). One- and two-way analysis of variance (ANOVA), Student’s t-test, and the Mann–Whitney U test were performed to determine significant differences. All statistical analysis was performed using GraphPad Prism v. 7.0.

## Results

### In silico *analysis of SEN1538 protein*

**Figure 1.**
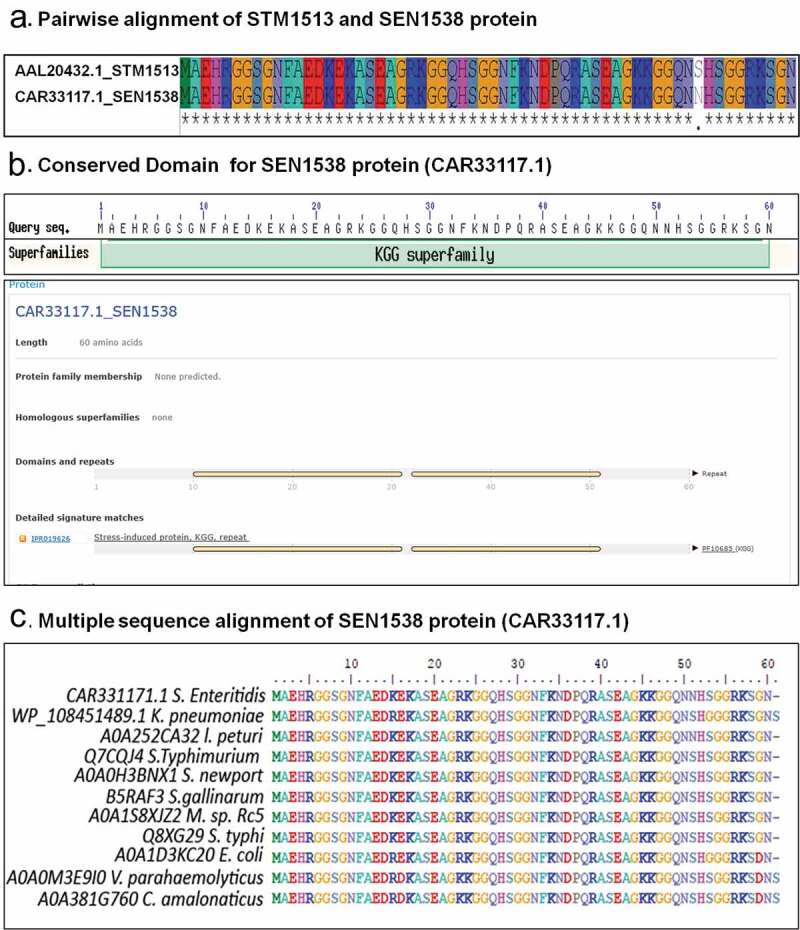
SEN1538 belongs to KGG superfamily of proteins. (a) Pairwise sequence alignment of STM1513 protein (GenBank Accession ID: AAL20432.1) from *Salmonella enterica* subspecies I serovar Typhimurium str. LT2 and SEN1538 protein (GenBank Accession ID: CAR33117.1) from *Salmonella enterica* subspecies I serovar Enteritidis str. P125109. (b) Superfamily and conserved domain of SEN1538 protein was screened by using NCBI Conserved Domain Tool and InterProScan5 Sequence Search Tool. (c) Multiple sequence alignment of KGG superfamily of proteins from different bacterial species against SEN1538 protein. *Salmonella* Enteritidis; *Klebsiella pneumoniae, Salmonella* Typhimurium; *Salmonella* Newport; *Salmonella* Gallinarum; *Micromonospora sp. Rc5, Salmonella* Typhi; *Escherichia coli; Vibrio parahaemolyticus; Citrobacter amalonaticus.*

Protein sequences of STM1513/STM14_1829 (GenBank Accession ID: AAL20432.1), YciG (GenBank Accession ID: AAL20646.1), and YmdF (GenBank Accession ID: AAL20052.1) were used as queries to find orthologs in *Salmonella enterica* subspecies I serovar Enteritidis str. P125109 (WT). Orthologs of YciG and YmdF were identified (Sequence identity: 100% and Sequence similarity: 100%; data not shown) and have been annotated the same as in *Salmonella enterica* subspecies I serovar Typhimurium. A protein encoded by *SEN1538* (GenBank Accession ID: CAR33117.1) was identified from genome of *Salmonella enterica* subspecies I Enteritidis str. P125109 that showed 98.3% sequence identity and 100% similarity (Figure 1(a)). Synteny analysis of the genomic region neighboring YciG, YmdF, and SEN1538 revealed the conservation of the neighboring genes across the two subspecies. *In silico* analysis of the YciG (data not shown), YmdF (data not shown), and SEN1538 proteins (Figure 1(b)) revealed that these belong to the KGG superfamily, corroborating with previous reports on their orthologs in *S*. Typhimurium. Proteins of the KGG superfamily are predicted to contain the stress-induced KGG repeat motif and the Walker A motif. In our study, we selected SEN1538 to further analyze its role in the bacterial stress response and virulence. This small protein is 60 amino acid residues (aar) in length, and was predicted to contain two stress-induced KGG repeat motifs from residue 10 to 30 aar and 32 to 50 aar (Figure 1(b)). However, the protein had no predicted Walker A motif. Moreover, SEN1538 was found to be conserved in several important bacterial pathogens, including *Escherichia coli, Klebsiella pneumonia, Salmonella* Typhi, and *Vibrio parahaemolyticus* (Figure 1(c)). As with the conserved KGG proteins in *S*. Typhimurium, SEN1538, YciG, and YmdF were identified as paralogs in *S*. Enteritidis.

### SEN1538 *promotes resistance to stressors*

The KGG protein superfamily was previously reported to consist of general stress proteins that play an important role in protection against acid stress, heat stress, bacterial virulence, and motility [17,20]. We therefore investigated the role of SEN1538 in the bacterial stress response under several environmental and intracellular stress conditions.

**Figure 2.**
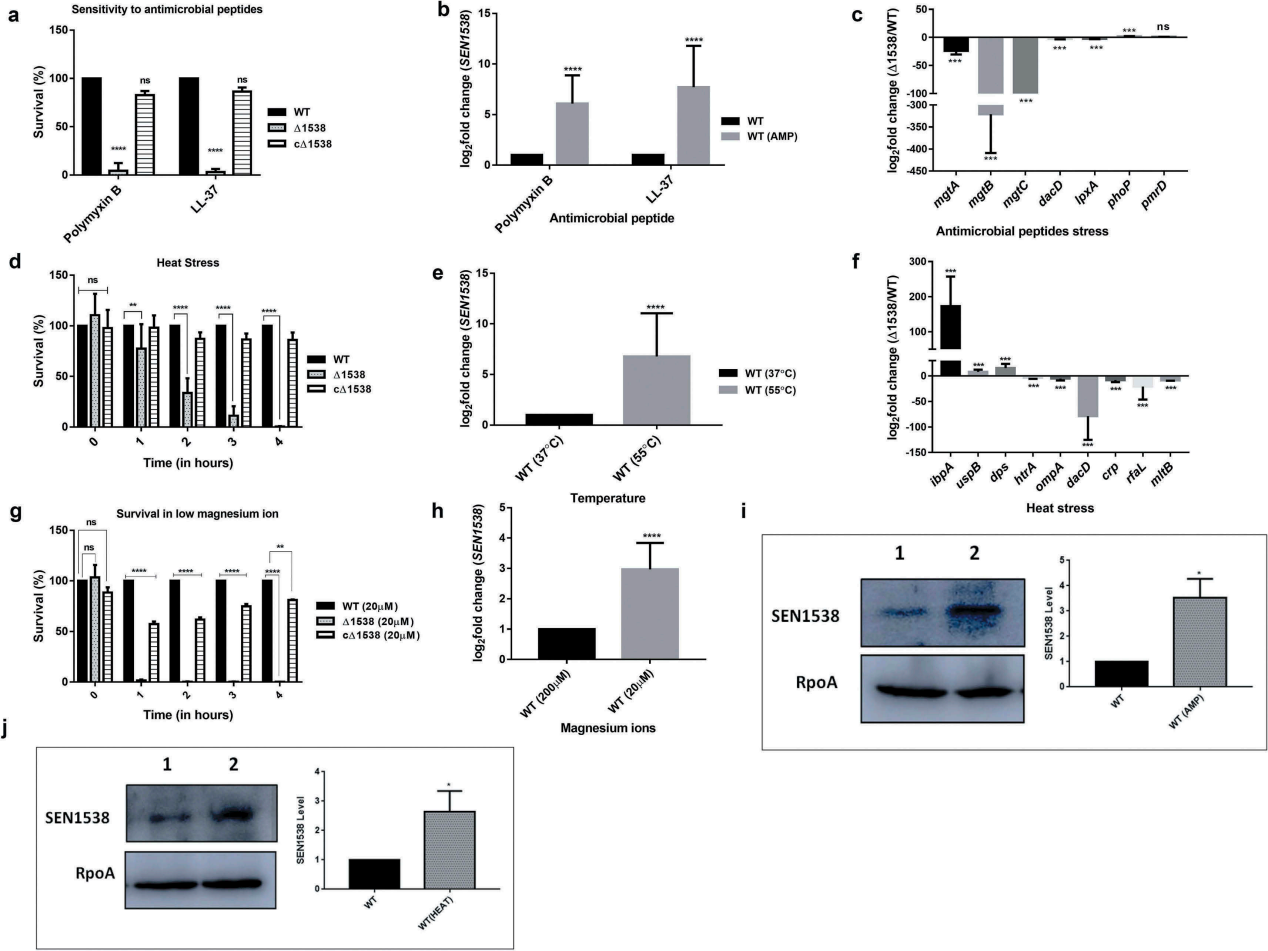
SEN1538 promotes resistance to stressors. The Log-phase cultures of Δ1538 and the complemented strain cΔ1538 were assessed under different stress conditions in comparison to WT (a) Antimicrobial peptides (AMPs) stress survival assay (b) Expression of *SEN1538* in WT after treatment with AMPs through qRT-PCR. (c) Expression of AMP resistance genes in Δ1538 compared to WT after treatment with PMB through qRT-PCR. (d) Heat stress survival assay at (55°C) (e) Expression of *SEN1538* in WT after exposure to heat stress through qRT-PCR. (f) Expression of stress response genes in Δ1538 compared to WT after exposure to heat stress through qRT-PCR. (g) Mg^2+^ starvation stress and survival assay (h) Expression of *SEN1538* in WT during Mg^2+^ starved condition. The bacterial counts were enumerated at indicated time points. Survivals (%) were compared to WT value normalized to 100%. *16 srRNA* gene was taken as housekeeping gene in qRT-PCR experiments. Protein levels of 6X-His tag fusion protein of SEN1538 in WT (WT-SEN1538His) strain were assessed during (i) AMP stress (PMB, 1 µg/mL) and (j) heat stress at 55°C by western blot and densitometry analysis. Western blots were scanned by GE Healthcare ImageQuant™ LAS 500 and band intensities were quantified by ImageJ^TM^ software. Lane 1, WT-SEN1538His (untreated); lane 2, WT-SEN1538His (stress). Each lane was independently compared to the control after normalization with loading control RpoA. Error bars indicate the mean±SD for three independent experiments. Statistical significance: *, P < 0.05; **, P < 0.01; ***, P < 0.001; ****, P < 0.0001; ns, not significant, P ≥ 0.05; Two-way ANOVA (Figure 2(a,d)); Student’s t-test Figure (2(b,c,e,f,h,j)).

WT, Δ1538, and the complemented strain cΔ1538 (see Table 1) were treated with the AMPs PMB (1 µg/mL) and LL-37 (10 µg/mL). Δ1538 had a survival count of 4.44% after treatment with PMB and 3.04% after treatment with LL-37 compared to WT (normalized to 100%) (Figure 2(a)). The complemented strain cΔ1538 showed survival restoration to 81% (PMB) and 86.2% (LL-37) compared to WT (Figure 2(a)). qRT-PCR analysis also showed a 6.08-fold increase in *SEN1538* expression after treatment with PMB, and 7.7-fold upregulation following treatment with LL-37 in WT (Figure 2(b)).

We also assessed transcript expression of genes that have been previously associated with resistance to AMPs [34,35]. qRT-PCR analysis showed significant 1.73-fold *phoP* upregulation and no change in *pmrD* expression (1.04-fold) in Δ1538 (Figure 2(c)). The upregulation of *phoP* in Δ1538 corroborated with a previous report on *phoP* upregulation in the mutant of ortholog STM14_1829 [19]. We further investigated the expression of PhoP-regulated genes in Δ1538, as these have been previously reported in stress resistance by *Salmonella* [34,36,37]. Genes encoding Mg^2+^ transporters *mgtA, mgtB*, and *mgtC* showed 23.73-fold, 320.77-fold, and 216.48-fold downregulation in Δ1538 compared to WT, respectively (Figure 2(c)). Furthermore, the LPS biosynthesis genes *dacD* and *lpxA* were downregulated 2.44-fold and 2.57-fold, respectively, in Δ1538 (Figure 2(c)). Interestingly, *phoP* upregulation did not drive the transcriptional activation of *mgtABC* genes in our mutant Δ1538. PhoP and its regulated genes such as Mg^2+^ transporters are activated in response to signals such as antimicrobial peptides, low pH, and low Mg^2+^ (≤40 µM) to promote survival inside the phagosomal environment of the host [35]. SEN1538 promoted resistance to AMPs, and its deletion led to significantly increased *phoP* expression in Δ1538. We, therefore, checked the survival of Δ1538 under other vacuolar stress signals. Compared to WT, Δ1538 strain deletion led to no difference in survival during acid stress at pH 3 ± 0.1 and pH 5 ± 0.2 (Figure S3A–S3B). Δ1538 had an attenuated survival (1.2%) in low Mg^2+^ conditions (Figure 2(g)). Furthermore, following culture in Mg^2+^-starved conditions, *SEN1538* showed a 3-fold increase in expression in WT compared to the control (Figure 2h). This showed that *SEN1538* did not confer resistance to acid stress and promoted survival under Mg^2+^-starved conditions.

Δ1538 was assessed for its survival during heat stress. After 1 h of exposure to high temperature at 55°C, Δ1538 had significantly reduced survival. Compared to WT (normalized to 100%), mutant survival decreased significantly from 33.67% at 2 h to 1% at 4 h after exposure to 55°C (Figure 2(d)). The complemented strain cΔ1538 showed survival restoration (80%) compared to WT (Figure 2(d)). *SEN1538* also showed a 6.78-fold increase in expression in WT following exposure to heat stress (Figure 2(e)), which corroborated with the reduced survival of Δ1538. We also checked the expression of several genes that have been previously associated with the heat stress response of *Salmonella* [10,38] in Δ1538 compared to WT. *htrA, ompA, dacD, crp, rfaL*, and *mltB* were downregulated from 3.75-fold to 78-fold in Δ1538 following exposure to high temperature at 55°C. In Δ1538, stress response regulators *ibpA, uspB*, and *dps* were also downregulated by 170-fold, 8-fold, and 15-fold, respectively, compared to WT (Figure 2(f)). Furthermore, following treatment with bile salts, the mutant showed an average survival rate of ~32% compared to WT (Figure. S3 C). Compared to the control, *SEN1538* was upregulated 15-fold in WT following treatment with bile stress (Figure S3D).

Western blotting with anti-His antibody showed significantly increased levels of 6X-His-tagged SEN1538 protein during AMP (Figure 2(i)) and heat stress (Figure 2(j)) in WT-SEN1538His (SEN1538 genome his-tagged in WT; see Table 1) compared to the control. Densitometry analysis of western blots showed 3.52-fold and 2.63-fold increases in SEN1538 after being exposed to AMP (Figure 2(i)) and heat stress (Figure 2(j)), respectively. Furthermore, in LB and M9 minimal medium, there was no difference in growth rate of the strains compared to WT. This suggests that the observed phenotypes were not due to growth defects (Figure S4A–S4B). These data, therefore, suggested that *SEN1538* was important for conferring resistance to different stresses.

### *RpoS regulates* SEN1538 *expression*

**Figure 3.**
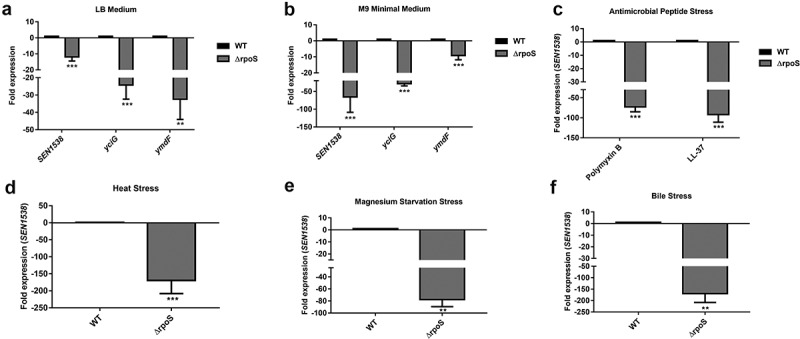
RpoS regulates *SEN1538* expression. WT and ΔrpoS were grown at 37°C at 150 rpm until log phase for subsequent experiments. Expression of *SEN1538, yciG* and *ymdF* in ΔrpoS compared to WT were checked under different conditions through qRT-PCR (a) LB medium (b) M9 minimal medium (c) Heat stress at 55°C (d) Antimicrobial peptide (AMP) stress (e) Mg^2+^ starvation stress (f) Bile stress. *16 srRNA* gene was taken as housekeeping gene for the analysis. Error bars indicate the mean fold expression ± SD. Statistical significance: **, P < 0.01; ***, P < 0.001; Student’s t-test.

The KGG family proteins YciG, YmdF, and STM1513/STM14_1829 are regulated by RpoS in *S*. Typhimurium and *E. coli* [14,16,39]. We investigated the role of RpoS in regulating *SEN1538* in *S*. Enteritidis. qRT-PCR analysis showed significantly reduced expression of *SEN1538, yciG*, and *ymdF* in ΔrpoS compared to WT when cultured in LB and M9 minimal medium (Figure 3(a,b)). *SEN1538* expression was further assessed in ΔrpoS under stress conditions. Compared to WT, the gene was downregulated in ΔrpoS by 73-fold and 91-fold following treatment with PMB and LL-37, respectively (Figure 3(c)). *SEN1538* was downregulated by ~150-fold following heat stress (Figure 3(d)). Compared to WT, the gene had ~77-fold reduced expression when cultured in Mg^2+^-starved conditions (Figure 3(e)), and ~169-fold downregulation following bile stress (Figure 3(f)) in ΔrpoS. Furthermore, *rpoS* showed significant ~20-fold to 60-fold increases in expression under these stress conditions (Figure S5A–S5D). This corroborated with the previously reported role of RpoS in the pathogen stress response. These data provide evidence that RpoS regulated *SEN1538* expression under normal and stressful conditions.

### *Δ1538 displays reduced invasion into epithelial cells* in vitro

**Figure 4.**
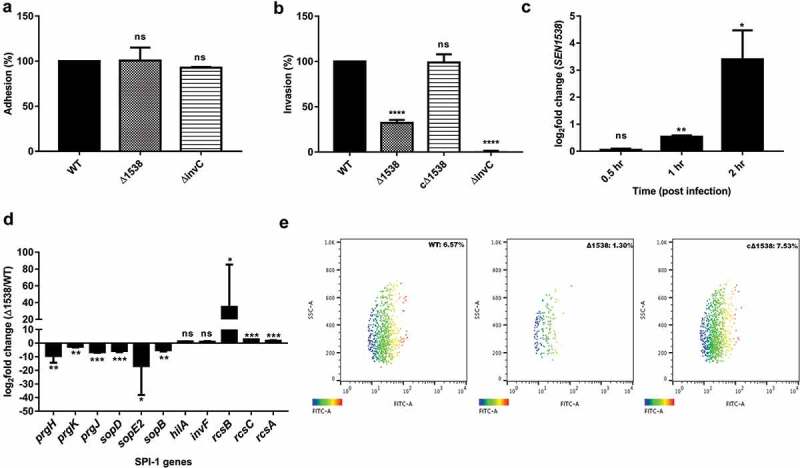
Δ1538 displays reduced invasion into HCT116 colon epithelial cells. Adhesion and invasion assay of WT, Δ1538 and the complemented strain cΔ1538 were performed in HCT116 cell line at MOI of 10. (a) Adhesion assays were performed on ice for 30 min. (b) Invasion assays were performed for 50 min. Noninvasive ΔinvC strain served as an experimental control. Data were represented as adhesion (%), invasion (%) for Δ1538, cΔ1538 and were compared to WT value normalized to 100%. (c) Expression of *SEN1538* in HCT116 after infection with WT at indicated time points through qRT-PCR. (d) Expression of SPI-1 genes in Δ1538 compared to WT through qRT-PCR. *16s rRNA* was taken as housekeeping gene in qRT-PCR analysis. Error bars indicate the mean ± SD of three independent experiments. (e) HCT116 cells were infected with GFP-expressing strains; WT-pCJLA, Δ1538-pCJLA and the complemented strain cΔ1538-pCJLA at MOI of 50. Invasion of strains was measured by the mean fluorescence intensity (MFI) of green fluorescent protein (GFP) expression (%). Data were acquired using BD FACScanto™ II cytometer (Becton–Dickinson, Erembodegem, Belgium) and analyzed by using Flowjo v. 10.4.2. Statistical significance: *, P < 0.05; **, P < 0.01; ***, P < 0.001; ****, P < 0.0001; ns, not significant, P ≥ 0.05; One-way ANOVA (Figure 4(a,b)); Student’s t-test (Figure 4(c,d)).

*S*. Enteritidis adheres to and then invades intestinal epithelial cells to initiate pathogenesis. Δ1538 showed no significant difference in its adherence to HCT116 colon epithelial cells compared to WT (Figure 4(a)). The mutant had a 67.8% lowered invasion in comparison to WT (100%) (Figure 4(b)). The complemented strain cΔ1538 showed invasion rate restoration (98%) compared to WT (Figure 4(b)). Furthermore, WT, Δ1538, and complemented strain cΔ1538 were transformed with the pCJLA plasmid for flow cytometery analysis, which was constitutively expressing GFP. The invasion rate of Δ1538-pCJLA (Table 1) into HCT116 was 1.30% compared to WT-pCJLA (6.57%) (Figure 4(e)). The complemented strain cΔ1538-pCJLA showed a restoration in invasion potential at 7.53% compared to WT-pCJLA (Figure 4(e)). We assessed *SEN1538* expression in WT after invasion into HCT116 at indicated time points (Figure 4(c)). The gene showed 10-fold and 68-fold increases in expression after 1 and 2 h of invasion into HCT116, respectively (Figure 4(c)).

Due to the reduced invasion of Δ1538 in HCT116 cells, the expression of SPI-1 genes was evaluated in Δ1538 compared to WT under SPI-1 inducing conditions. The SPI-1 genes encoding for type three secretion system (T3SS1) assembly were *prgH, prgK*, and *prgJ*, which were downregulated 9.37-fold, 2.63-fold, and 6.69-fold in Δ1538, respectively. The T3SS1 effector *sopD* was downregulated by 5.77-fold in Δ1538 compared to WT. Additionally, *sopE2* and *sopB* had respective decreases in expression of 16.85-fold and 5.14-fold in Δ1538 (Figure 4(d)). The negative regulators of SPI-1 *rcsB* (34.8-fold), *rcsC* (2.67-fold), and *rcsA* (1.77-fold) were upregulated in the mutant. These findings, therefore, provided evidence for the role of *SEN1538* in *S*. Enteritidis invasion.

### SEN1538 *promotes survival within macrophages* in vitro

**Figure 5.**
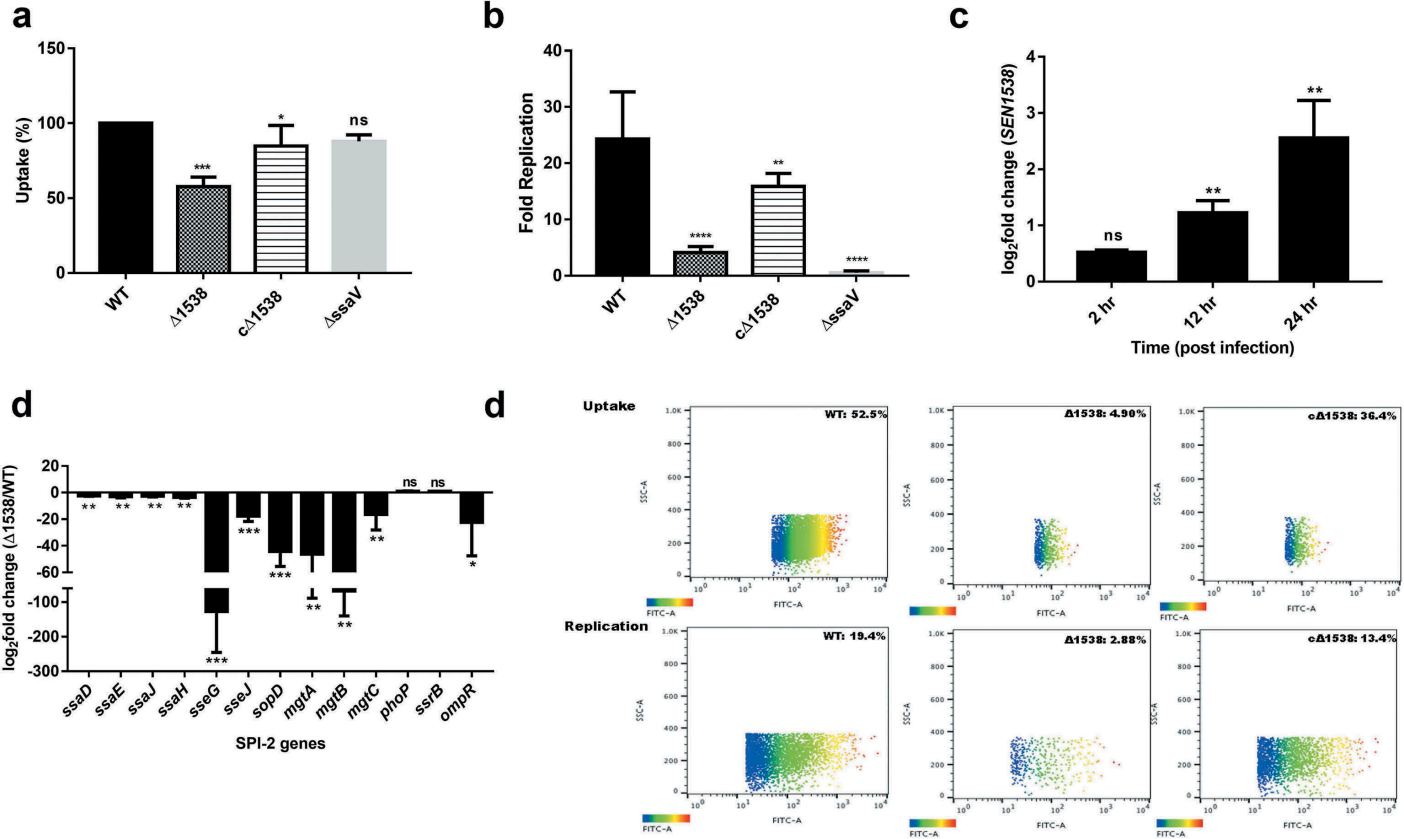
*SEN1538* promotes survival within RAW264.7 murine macrophage cells. RAW264.7 cell lines were infected with WT, Δ1538 and the complemented strain cΔ1538 at MOI of 10 for (a) Macrophage uptake assay and (b) Intra-macrophage survival assay. Bacterial counts were enumerated by serial dilution and plating at 2 h and 24 h of infection. Uptake (%) for Δ1538 and cΔ1538 was compared to WT (normalized to 100%). Intra-macrophage survival was represented in fold survival and compared to WT. ΔssaV defective for intra-macrophage survival serves as the experimental control. (c) Expression of *SEN1538* in RAW264.7 after infection with WT at indicated time points through qRT-PCR. (d) Expression of SPI-2 genes in Δ1538 compared to WT through qRT-PCR. *16s rRNA* was taken as housekeeping gene in qRT-PCR experiments. Error bars indicate the mean ± SD of three independent experiments. (e) Validation of macrophage uptake and survival assay was done by flow cytometry. RAW264.7 cells were infected with GFP-expressing strains; WT-pCJLA, Δ1538-pCJLA and the complemented strain cΔ1538-pCJLA at MOI of 50. Data were acquired at 2 h (uptake) and 24 h (replication survival) using BD FACScanto™ II cytometer (Becton–Dickinson, Erembodegem, Belgium) and analyzed by using Flowjo v. 10.4.2. Statistical significance: *, P < 0.05; **, P < 0.01;***, P < 0.001; ****, P < 0.0001; ns, not significant, P ≥ 0.05; One-way ANOVA (Figure 5(a,b)); Student’s t-test (Figure 5(c,d)).

Δ1538 had a 40% decreased uptake in the RAW264.7 murine macrophage cell line compared to WT (Figure 5(a)). The mutant also displayed a 5-fold decrease in intra-macrophage survival compared to WT at 24 h p.i in the RAW264.7 cell line (Figure 5(b)). The complemented strain cΔ1538 showed 3.5-fold increased survival compared to the mutant, indicating the partial restoration of its phenotype. Flow cytometry also demonstrated that Δ1538-pCJLA had a 4.9% reduced uptake compared to WT-pCJLA (52.5%). The mutant had attenuated survival (2.88%) at 24 h p.i in RAW264.7 cells compared to WT-pCJLA (19.4%) (Figure 5(e)). The complemented strain cΔ1538-pCJLA showed restored uptake and survival phenotype compared to WT-pCJLA (Figure 5(e)). In RAW264.7 cells, *SEN1538* had 2.45-fold and 5-fold increased mRNA expression in WT at 12 and 24 h p.i., respectively (Figure 5(c)). This indicated the possible role of *SEN1538* in the intra-macrophage survival of *S*. Enteritidis.

Due to decreased Δ1538 survival inside macrophages, SPI-2-encoding T3SS-2 genes and SPI-2 effector genes were evaluated in the mutant compared to WT in SPI-2-inducing medium (Figure 5(d)). We observed downregulation of the T3SS-2 assembly genes *ssaD* (2.4-fold), *ssaE* (3.06-fold), *ssaJ* (2.771-fold), and *ssaH* (3.7-fold) in Δ1538 compared to WT. The SPI-2 effectors *sseG, sseJ*, and *sopD* had 127.12-fold, 17.68-fold, and 44.26-fold reduced expression in Δ1538 compared to WT, respectively (Figure 5(d)). Mg^2+^ transporters are important activators of the SPI-2 and sensor kinase system [40] and were checked for expression in Δ1538. Compared to WT, mutant *mgtA, mgtB*, and *mgtC* genes showed a pronounced reduction in expression by ~46-fold, 67-fold, and 17-fold, respectively (Figure 5(d)). SPI-2 regulators such as *phoP* and *ssrB* displayed no significant changes in expression, while *ompR* was downregulated by 20-fold in Δ1538. These findings supported the role of *SEN1538* in *S*. Enteritidis survival in phagocytic cells.

### SEN1538 *deletion impairs systemic survival and colonization* in vivo

**Figure 6.**
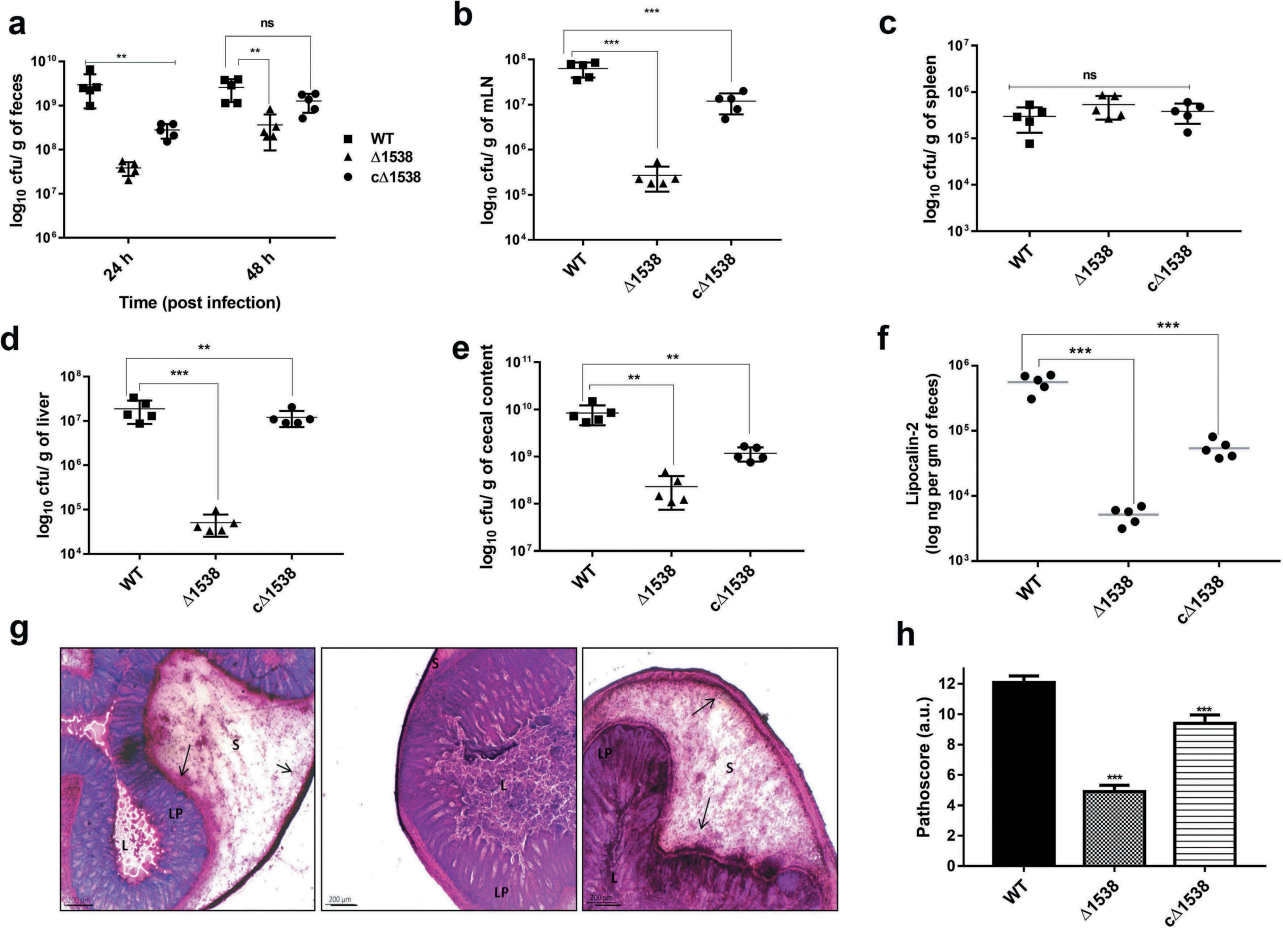
*SEN1538* deletion impairs systemic survival and colonization *in vivo*. Streptomycin-treated C57BL/6 mice (n = 5) were infected with ~10^7^ cfu of WT, Δ1538, the complemented strain cΔ1538 and PBS (negative control) by oral gavage. (a) Bacterial loads in the feces were enumerated at 24 h and at 48 h post-infection (p.i.). (b-e) Organ loads infected with WT, Δ1538 and cΔ1538 were enumerated at 72 h p.i. Mice groups were euthanized and the bacterial load in organs (b) mesenteric lymph node (mLN) (c) spleen (d) liver and (e) cecal content were determined. (f) Lipocalin-2 concentrations from mice feces supernatant were monitored by ELISA. Bar indicates median. (g) Hematoxylin & Eosin (H&E)-stained representative cecum tissue sections (size: 5 µM) from each group of mice showing induced cecal inflammation from WT, Δ1538, and cΔ1538 (left to right). L, lumen; LP, lamina propria; S, submucosal edema. Bars, 200 µM. (h) Cecal pathoscores were obtained by examining the H&E stained cecal tissue sections from each mouse of all groups. Data were represented as mean ± SD. Statistical significance: ns, not significant P ≥ 0.05; *P < 0.05; **P < 0.01; ***P < 0.001; Mann–Whitney U test (Figure 6(a,b,c,d,e,h)); One-way ANOVA (Figure 6(f)).

A C57BL/6 mouse model of systemic infection was used for assessing the virulence of Δ1538 (n = 5). Mutant Δ1538 displayed reduced bacterial load in feces by 2-fold at 24 and 48 h p.i. compared to WT. The complemented strain cΔ1538 showed a 1-fold increase in the fecal bacterial count relative to Δ1538 (Figure 6(a). The bacterial load in mLN, spleen, liver, and cecal contents were enumerated at 72 h p.i. Δ1538 showed a 3-fold reduction in colonization in mLN, liver, and cecal contents compared to mice infected with WT (Figure 6(b–e)). Complementation of *SEN1538* in Δ1538 partially rescued colonization in mice. cΔ1538 had 3-fold higher survival in mLN and liver and 1-fold higher survival in the cecal contents compared to the mutant (Figure 6(b–e)). Gut inflammation was measured via estimation of lipocalin-2 levels in feces. The mean lipocalin-2 concentration elicited by mice infected with Δ1538 was 2-fold lower than WT. The complemented strain cΔ1538 elicited 1-fold higher lipocalin-2 response than Δ1538, which partially rescued the inflammatory phenotype (Figure 6(f)). Moreover, histopathological evaluation of WT- and cΔ1538-infected mice showed pronounced cecal inflammation, while cecum infected with Δ1538 displayed significantly reduced inflammation (Figure 6(g)). Δ1538 had a low pathoscore of 5 compared to those of WT and cΔ1538 (12.08 and 10, respectively) (Figure 6(h)). *Salmonella*-induced inflammation was further validated via ELISA, which was performed to estimate serum levels of the cytokines *TNF-α, IL-6, IL1-β*, and *IFN-γ*, and the two chemokines *Kc* and *MIP-2* (Figure S7). Δ1538 elicited significantly reduced levels of these cytokines and chemokines compared to WT. The reduced lipocalin-2 and cytokine levels in Δ1538-infected mice groups were in congruence with the mutant attenuated survival in the gut lumen and systemic organs of mice. In conclusion, *SEN1538* was essential for the systemic survival of *S*. Enteritidis and its colonization inside the host.

### Global changes in the Δ1538 transcriptome compared to WT

**Figure 7.**
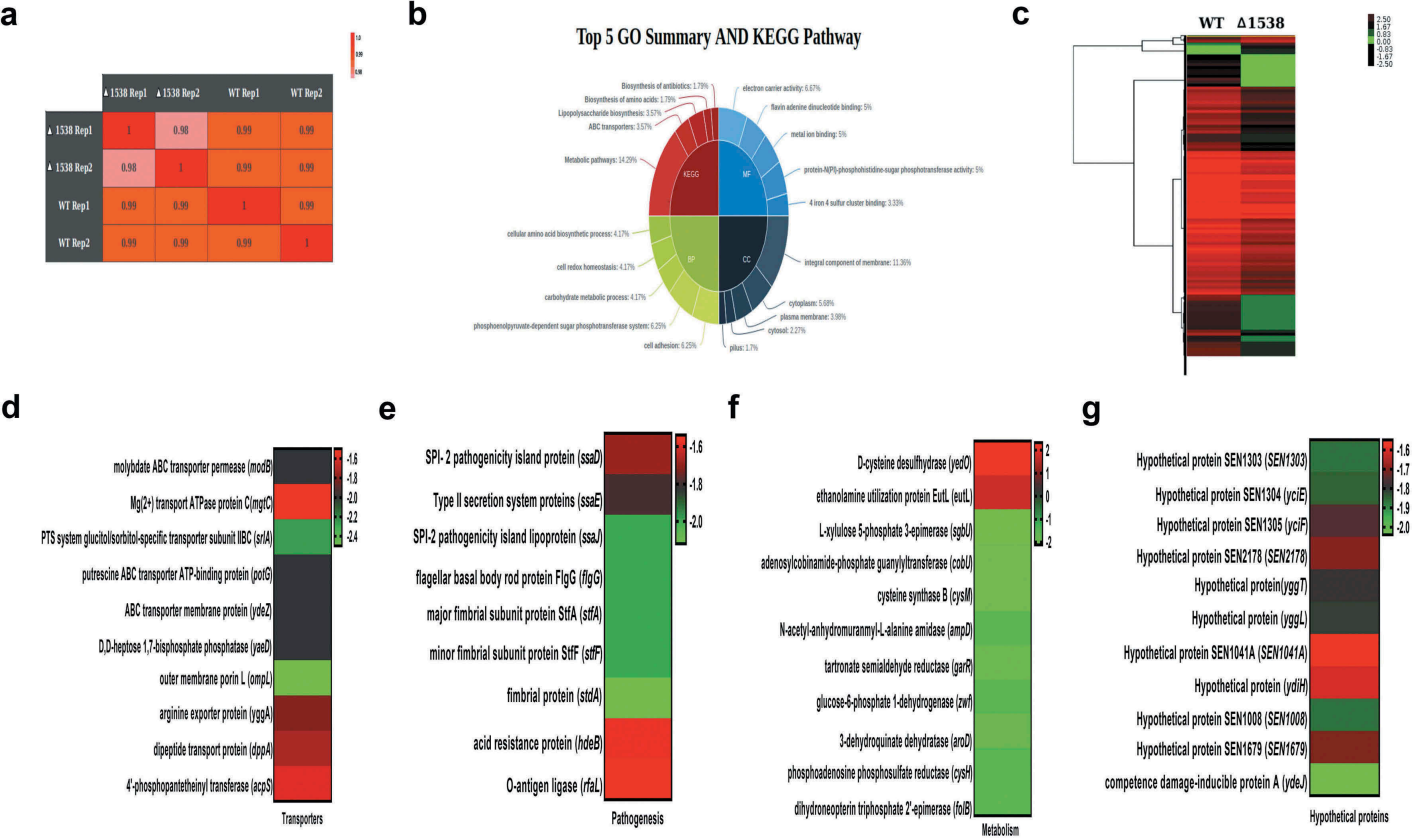
Global changes in the Δ1538 transcriptome compared to WT. (a) Sample replicates of Δ1538 and WT displayed a high degree of correlation with Pearson correlation coefficients ranging from 0.98 to 1. (b) Pie-Donut depicting the top 5 GO and KEGG Pathway illustrating functional clusters that are differentially expressed in Δ1538 against WT. (c) Hierarchical clustering of differentially regulated genes showing sample duplicates of WT and Δ1538. (d-g) Expression profiles of differentially regulated genes in Δ1538 compared to WT, clustered according to their functions. (d) Transporters (e) Pathogenesis (f) Metabolism (g) Hypothetical proteins. Transcriptome data were analyzed and differentially expressed transcripts were selected on the basis of reads per kilobase per million mapped sequence reads (RPKM) >1 in either of the pair of samples with Log_2_ fold change ≤ −1.5 for downregulated genes and Log_2_ fold change ≥1.5 for upregulated genes. Statistical significance: t-test P-Value <0.05.

Comparative global transcriptomic analysis of Δ1538 was conducted by measuring the mean expression of sample duplicates across the two strains, using an Illumina HiSeq4000 sequencing platform. The goal was to survey the global changes in gene expression associated with SEN1538 deletion. We also aimed to decipher the role of these differentially expressed genes in the attenuated survival of Δ1538 during infection and stressful conditions. Approximately 4206 transcripts were identified after alignment, from which 3195 genes were identified and annotated. There was a high degree of correlation within the sample duplicates of each strain, with Pearson coefficients (*r*) of 0.98 and 0.99 for Δ1538 and WT, respectively (Figure 7(a)). A total of 111 genes were identified as differentially regulated in Δ1538 compared to WT, with a Log_2_ fold change cutoff of ≥1.5 (for upregulation) or ≤ −1.5 (for downregulation) (P < 0.05). Among these genes, 103 were downregulated and 8 were upregulated in Δ1538. A complete list of transcripts identified through RNA-seq, the differentially expressed genes, and other associated information are provided in Dataset S1. The global changes in gene expression among the strains are shown in the form of a heat map (Figure 7(c)). Functional annotation clustering was performed using the GO and KEGG metabolic pathways. GO analysis revealed a cluster of genes enriched to cell adhesion (6.25%) and integral components of the membrane (11.36%) based on their biological processes and cellular components, respectively. KEGG pathway analysis showed 14.29% genes enriched for metabolic pathways (14.29%), transporters (3.57%), and lipopolysaccharide (LPS) biosynthesis (3.37%) (Figure 7(b)). Representative heat maps of differentially expressed genes belonging to metabolism, transport, pathogenesis, and hypothetical genes are shown in Figures 7(dg).

To validate or dispute the current transcriptome data, 23 differentially expressed (18 downregulated, 5 upregulated) genes were selected based on the major functional clusters identified in the study. qRT-PCR and RNA-seq data correlated well with variation in their fold change values, which may have been due to differences in the sensitivities of the two platforms for RNA analysis (Figure S8).

## Discussion

SEN1538 protein and its paralogs YciG and YmdF belong to a family of small proteins with tandem repeats of KGG motifs. The orthologs of these proteins YciG, STM14_1829, and YmdF were predicted and confirmed to be intrinsically disordered proteins (IDPs) that remain unstructured in solution [19]. Recent studies have predicted that the three paralogous proteins YciG, YmdF, and STM1513/STM14_1829 modulate the flagellum-dependent motility of *S*. Typhimurium [19]. YciG mutants showed defective motility compared to its parent strain *S*. Typhimurium SB300, and STM1513/STM14_1829 mutants showed defective motility compared to its parent strain S. Typhimurium ATCC14028. However, we did not observe any defect in the swimming motility of deletion strains Δ1538, ΔyciG, and ΔymdF compared to WT (see Figure. S6). The genetic differences between these species of *Salmonella* could possibly account for these discrepant phenotypes. Studying the phenotypes of Δ1538 and ΔymdF in *S*. Typhimurium SB300 would be an interesting future avenue for research.

The KGG motif has not yet been functionally characterized, but it is ubiquitously conserved in stress-inducible proteins across several Gram-negative pathogens of the *Enterobactericae* family. RpoS sigma factor has been known to regulate the activation of several stress-inducible proteins, such as the KGG family, in response to varied stress signals like pH, temperature, AMPs, nutrient starvation, and reactive oxygen and nitrogen species [1,13,41]. Previous reports on RpoS mutants have shown them to have defective swimming motility and phagocytic survival, be sensitive to stress, and be avirulent inside a host [14,42,43]. YciG, STM14_1829, and YmdF expression was shown to be regulated by RpoS [14–16,21,39,44,45]. A promoter specificity assay has also detected a RpoS binding site in the promoter region of these genes [15,21]. Global transcriptomic and proteomic profiling studies of the *rpoS* mutant in *E. coli* [46,47] and *S*. Typhimurium showed *yciG, ymdF*, and *STM14_1829/STM1513* downregulation [14,39,43]. Our study also showed decreased expression of *SEN1538, yciG*, and *ymdF* in ΔrpoS in LB medium, M9 minimal medium, and under stress conditions. Interestingly, YciG in *E. Coli* and *S*. Typhimurium was reported to confer protection against thermal and acid stress [20,21,48]. The *Salmonella* YciG deletion mutant was also reported to have impaired phagocytic survival, reduced invasion, and reduced adhesion onto host epithelial cells, suggesting that YciG has a role in pathogen virulence. Of the three paralogs, we chose SEN1538 to functionally decipher its role in the bacterial stress response and virulence of *S*. Enteritidis.

Our study reports that SEN1538 is an important stress protein for bacterial survival during heat stress, AMP stress, Mg^2+^ starvation stress, and bile stress. Unlike YciG, SEN1538 did not confer protection against acidic conditions (pH 3 ± 0.1 and pH 5 ± 0.2) in *S*. Typhimurium and *E. coli* [17,20,21]. Δ1538 survival was also assessed in the presence other host stress factors such as oxidative stress (0.03% H_2_O_2),_ nitrosative stress (750 µM spermine NONOate), and nutrient limitation (glucose starvation; 0.04% glucose). These are important environmental signals to trigger virulence genes in the vacuolar compartments of host macrophages [42]. The mutant displayed no difference in survival compared to WT under these conditions (data not shown).

Many genes that were previously reported to be associated with the heat and AMP stress response of *Salmonella* were downregulated in Δ1538 in our study. These included *htrA, ompA, dacD, crp, mltB*, and *rfaL* [1,10,49]. Strains defective for survival in low Mg^2+^ conditions showed hypersusceptiblity to the action of AMPs [36]. Furthermore, an increased accumulation of Mg^2+^ has been shown to have a role in thermotolerance by protecting membrane integrity and proteins in *S*. Typhimurium [9,50]. One of the key observations of our study was the downregulation of Mg^2+^ transporters encoded by *mgtABC*. These maintain Mg^2+^ homeostasis to facilitate membrane modifications in response to vacuolar stress, and signal the transcriptional activation of several SPI genes during infection [34]. This explained Δ1538 showing a sensitized phenotype to several stress factors, which are likely to be encountered during host infection. Δ1538 displayed reduced survival under bile stress, corroborating with the previously reported role of the KGG family protein YciG [16,51,52].

Δ1538 had reduced invasion into HCT116 cell lines due to the augmented expression of SPI-1 genes, which facilitate attachment and entry into the host epithelium. An *in vitro* epithelial cell model showed an AMP-sensitive *Salmonella* mutant had reduced invasion into epithelial cell lines [53]. Similarly, a heat-sensitive *htrA* mutant was reported to be less virulent in mice and displayed reduced invasion across epithelial cells [54]. Δ1538 showed decreased survival in RAW264.7 phagocytic cell lines. This could be due to its sensitivity to AMPs and low Mg^2+^ conditions, which are likely to be encountered inside the vacuolar compartments of phagocytes. Mutants of AMP resistance genes were shown to have reduced intracellular replication in professional phagocytes and augmented SPI-2 expression [55,56]. Our findings were in accordance with the previously reported phenotype of the pathogenic stress response [20].

Δ1538 had reduced counts in feces and attenuated systemic survival in the mLN, spleen, and cecum of C57BL/6 mice. This could be due to reduced survival of Δ1538 in the gut lumen, which may be attributed to clearance of the mutant load at Peyer’s patches, the oral cavity, or intestinal epithelia enriched with Cathelin-related antimicrobial peptide, an ortholog of human LL-37 (CRAMPS), and resident macrophages. A similar outcome was observed in *yejF* and *pmrHFIJKLM* mutants, which are also involved in PMB resistance in *S*. Typhimurium [56,57].

*Salmonella* utilizes gut mucosal inflammation for successful host colonization [23]. Accordingly, impaired Δ1538 survival in mice elicited reduced levels of fecal lipocalin-2, which serves as biomarker of intestinal inflammation [23].

The composition of RpoS is dynamic, and the expression of genes under this regulon changes greatly in response to different environmental signals. In our study, *SEN1538* exhibited phenotypes and influenced the expression of various genes under specific stress and infection conditions. This is similar to previously reported observations in *rpoS* mutants [14,16]. This could be a result of an epistatic interaction with RpoS or pleotropic regulation due to genomic deletion of *SEN1538*. Moreover, most of the differentially expressed genes in this study belonging to metabolism, pathogenesis, and transporters were also differentially regulated in the previously reported *rpoS* mutant of *S*. Typhimurium [14,16]. Our transcriptomic analysis reported downregulation of several ABC transporters such as *dppA, modB, potG, cysW, cysH, and cysM* in Δ1538. ABC transporters like *sapABCDF* and *yejABEF* were previously reported to contribute to the AMP resistance and virulence of *S*. Typhimurium [56,58]. *cysAUW, yfeABCD*, and *sitABCD* were required for the virulence of *Yersinia pestis, S*. Typhimurium, and *Bacillus subtilis* in mice [58]. A subset of genes (14%) involved in amino acid and central energy metabolism were differentially regulated in Δ1538 compared to WT. Amino acid auxotrophs and carbon metabolic mutants of *aroA, aroD*, and *zwf* in *S*. Typhimurium were previously reported to have a defective cell wall, be highly sensitive to antimicrobials, and have attenuated virulence *in vivo* [59]. RNA-seq analysis of Δ1538 suggested an extensive remodeling of these pathways in pathogens in order to adapt to different conditions [14]. On the basis of the above findings, we hypothesize that the stress sensitivity and virulence defects of *rpoS* mutants of *Salmonella* may be partly attributed to lack of *SEN1538* expression.

An ortholog of SEN1538 and its paralogs were previously reported as IDPs [19] in *S*. Typhimurium. IDPs recognize and interact with various molecules, such as proteins, nucleic acids, and transcriptional regulators [60]. These interactions regulate diverse cellular pathways in bacteria [60]. One of the major observations in this study was the downregulation of Mg^2+^ transporters encoded by *mgtABC*. This gene mediates Mg^2+^ homeostasis that stimulates membrane modifications in response to vacuolar stress. Several SPI genes are subsequently transcriptionally activated during infection, especially inside phagosomes [34]. We hypothesized that *SEN1538* interacts with regulators of Mg^2+^ transporters. This interaction may enable membrane resilience to stress and serve as a checkpoint to trigger the expression of virulence genes required for systemic survival. Our data highlight some unanswered questions about the mechanisms underlying the diverse roles of *SEN1538* during stress and infection. Thus, there should be focused studies addressing these vital problems in the future. In conclusion, our findings may help develop new therapeutic interventions useful for industrial and clinical applications to control *Salmonella* infection.

## Figures and Tables

**Table:** 

AMP	Antimicrobial peptides
CRAMPS	Cathelin-Related Antimicrobial Peptide
DMEM	Dulbecco’s modified Eagle Medium
ELISA	Enzyme Linked Immunosorbent Assay
FBS	Fetal Bovine Serum
GFP	Green Fluorescent Protein
GO	Gene Ontology
HE	Hematoxylin–Eosin
IDP	Intrinsically Disordered Proteins
KEGG	Kyoto Encyclopedia of Genes and Genomes
LB	Luria bertani media
LPS	Lipopolysaccharide
MOI	Multiplicity of infection
O.D.	Optical density
P.I.	Post infection
PBS	Phosphate buffered saline
PMB	Polymyxin B
qRT-PCR	Quantitative Real Time Polymerase chain reaction
RPKM	Reads Per Kilobase Per Million
SCV	*Salmonella* Containing Vacuole
SPI	*Salmonella* Pathogenicity Island
T3SS	Type Three Secretion System
WT	Wild Type

## Supplementary material

The supplementary data for this article can be accessed here.
