# Supplementary material for: RpoS-regulated SEN1538 gene promotes resistance to stress and influences Salmonella enterica serovar enteritidis virulence
Source: Virulence. 2020 Apr 5;11(1):295–314. doi: 10.1080/21505594.2020.1743540 (PMC7161692; doi:10.1080/21505594.2020.1743540)
Supplement: Supplemental Material [file kvir-11-01-1743540-s001.docx]

**RpoS Regulated *SEN1538* Gene Promotes Resistance to Stress and Influences *Salmonella enterica* serovar Enteritidis Virulence. Arunima *et al*. 2019.**

**SUPPLEMENTARY TABLE**

**Table. S1. List of primers used in the study**

| **Table. S1. List of primers used in the study** | |  |
| --- | --- | --- |
| **Knockout Primer** |  | **References** |
| **Gene** | **Sequence (5' to 3')** |  |
| Fw1538 KO | ATGTGCAATATTTCAGAATATATTTACGAAATATCAGGAGTAAAAGTGTAGGCTGGAGCTGCTTC | This study |
| Rw1538KO | AGACATATCATATTTAAAACGCAACATCATCATGAGGATTATATTCATATGAATATCCTCCTTAG | This study |
| FwrpoSKO | AAAAAAAGGCCAGTCTGTCGACTGGCCTTTTTTTGACAAGGGTACGTGTAGGCTGGAGCTGCTTC | This study |
| RwrpoSKO | TTTGACTTGCTAGTTCCGTCAAGGGATCACGGGTAGGAGCCACCTTCATATGAATATCCTCCTTAG | This study |
| FwyciGKO | CAGATTGCTTGATAAAAGCATGTGTTGTATTTACATTACAGTAAAGTGTAGGCTGGAGCTGCTTC | This study |
| RwyciGKO | GTTAAAACCGATTTTTTCAGCAAGCAACGAGACAGGAGAAATAATACATATGAATATCCTCCTTAG | This study |
| FwymdFKO | CATTACCTATGCAATATGATGTCTAATCTATGACGGAGGTCAGTAGTGTAGGCTGGAGCTGCTTC | This study |
| RwymdFKO | GCTTTTTAGCGCGAAACGCTCAGCGTCTTGCTGTTGTGACGCAGGCATATGAATATCCTCCTTAG | This study |
| Confo 1538 | TATTAGAAAAATGCGTACACTTCGC | This study |
| Confo rpoS | GTCAGCATTGTCTGTATACCT | This study |
| Confo yciG | TAGCCAGAGCCACGTCCAAT | This study |
| Confo ymdF | ACTTATTTATTTCTCATTGTTA | This study |
| Km/Kt | CGGTCCGCCACACCCAGCC | This study |
| **Primers for Chromosomal his tagging** |  |  |
| FwSEN1538-His | TGGCCAGAATAACCACAGTGGCGGACGGAAATCCGGCAATCATCATCACCACCACCATTAAGTGTAGGCTGGAGCTGCTTC | This study |
| RwSEN1538-His | ATATCATATTTAAAACGCAACATCATCATGAGGATTATATTTTACATATGAATATCCTCCTTAG | This study |
| Confo SEN1538-His | GCAATCATCATCACCACCACCAT | This study |
| **Primer For complementation (cloning primer)** | |  |
| **Gene** | **Sequence (5' to 3')** |  |
| 1538Pch_Nco (Fw) | CATGCCATGGGGGACGTTATTACGCTCTTCCATG | This study |
| 1538Pch_Xba (Rw) | GCTCTAGATTAATTGCCGGATTTCCGTCC | This study |
| **Primers for qRT-PCR** |  |  |
| **Gene** | **Sequence (5' to 3')** |  |
| SEN1538Fw | GTCCGCCACTGTGGTTATTC | This study |
| SEN1538Rw | CGGCGGTTCCGGTAATTTT | This study |
| pmrDFw | AAGGTCTGGTGTTTTTCCCGA | This study |
| pmrDRw | TCAAAAAGAGGGCGTGCCA | This study |
| mgtAFw | CGCTAAAAGCGATGGTGAGC | This study |
| mgtARw | TAATCCGTAAATCCGCCGGG | This study |
| mgtBFw | ATTTTACTGCGTGAAGCGGC | This study |
| mgtBRw | TTCATAGCCCGGAGGAGACA | This study |
| mgtCFw | GGGACCGAACCTAACCCTTG | This study |
| mgtCRw | ATTTTACTGCGTGAAGCGGC | This study |
| rcsAFw | GTCAGCCGCACTACGTGATA | This study |
| rcsARw | CAGCATGTTGCGAATGTGGA | This study |
| rcsBFw | GGGATCGTACTCAAGCAGGG | This study |
| rcsBRw | ACTTCGCTCTCTTTTGGCGA | This study |
| rcsCFw | CGAGTCCTTCCACGCTCTTT | This study |
| rcsCRw | CCTTCTTCCAGGTGGGAACC | This study |
| ibpAFw | CTCGGCAATGCCCTGATACA | This study |
| ibpARw | AGCCAAAGTAATGGCGGCTA | This study |
| dpsFw | CGCTCGGCCATAGTATCCAG | This study |
| dpsRw | AAAAGCGACGGTTGAGTTGC | This study |
| uspBFw | GCGTTCACAACGGCGAATAA | This study |
| uspBRw | ACTTCGTGGTTGCGATCCTT | This study |
| ibpAFw | CTCGGCAATGCCCTGATACA | This study |
| ibpARw | AGCCAAAGTAATGGCGGCTA | This study |
| ompAFw | GCGAGGTTTCACGTTGTCAC | This study |
| ompARw | TCGTTCTGGGCTTCACTGAC | This study |
| dacDFw | ACCGCCGATGCGATAAGATT | This study |
| dacDRw | GCGATTATTCACGGCGAACC | This study |
| crpFw | GAAAACCGCATGTGAGGTCG | This study |
| crpRw | GTTTCGCCAGATTCAGCAGC | This study |
| rfaLFw | GTGCTTAGCGCCATCTACCT | This study |
| rfaLRw | AGTGATAACCAAAGCGCCGA | This study |
| mltBFw | ATCGCCTTTCACCCAACCAT | This study |
| mltBRw | AGCAAGACGATCCGCTCAAT | This study |
| lpxAFw | GAATCGCTGTATCCTCGCCA | This study |
| lpxARw | CATACGGAGGGACATCCTGC | This study |
| htrA Fw | GAGTGCACTGGCTCTGAGTT | This study |
| htrA Rw | TTCACCGTGGTGCTACCTTC | This study |
| prgHFw | GCCAGCTGCGGATAATAGGT | This study |
| prgHRw | CGTTTTCAGGTGTTGCCAGG | This study |
| prgKFw | GCCTGCTGACATCACGGATA | This study |
| prgKRw | TGCGCATCAGATCAGCGATA | This study |
| prgJFw | GAATCGCTGTATCCTCGCCA | This study |
| prgJRw | CATACGGAGGGACATCCTGC | This study |
| sopDFw | TGGTTCGAAGATGACCTGGC | (1) |
| sopDRw | AGTGAGTCCTGCCATTCGAC | (1) |
| sopE2Fw | CCTTTTGTCGTCCCCCTCAT | This study |
| sopE2Rw | CGTTGGCATCGTCCCCTTAT | This study |
| sopBFw | GAAGACTACCAGGCGCACTT | (2) |
| sopBRw | GATGGCGGCGAACCCTATAA | (2) |
| hilAFw | GGGCAGATGATACCCGATGG | (1) |
| hilARw | AAGAGAGAAGCGGGTTGGTG | (1) |
| invFFw | TCCACTAATCCTGCGCCATC | (1) |
| invFRw | CCGTTGTCGCACCAGTATCA | (1) |
| ssaDFw | CGCTTGACGAGCAATAACCG | This study |
| ssaDRw | TGGTTGGCTGGCGTATTCTT | This study |
| ssaEFw | AGTGCGCTGTTATGGTAACGA | This study |
| ssaERw | CTTCATTCGCGTGAAGAGGC | This study |
| ssaJFw | TGCATTAATCACGCCCTCCA | This study |
| ssaJRw | AACGGTTATCCGCATCGTCA | This study |
| ssaHFw | AAATAACAGACGCAGCGCCA | This study |
| ssaHRw | CGGGCGTTAACCATAGCCT | This study |
| sseJ Fw | GGACTTCCTGGGTTGGATGG | This study |
| sseJ Rw | AAAGCATCGCTCACAATGCC | This study |
| sseG Fw | GGTCATTGCCATCCCATCCA | This study |
| sseG Rw | TTGCCTATGGCTCACGACAG | This study |
| phoP Fw | CACTGCCGGTTCTGGTGTTA | This study |
| phoP Rw | GATCACTTGGGAGGCCAGAC | This study |
| ssrB Fw | ACGCTGACACGACCAATCAT | This study |
| ssrB Rw | CGGTGTGTTTCGACGGTTTT | This study |
| ompRFw | TTCAGTACCGCAAACTCCCC | This study |
| ompRRw | TACGTCGTCAGGCAAACGAA | This study |
| 16sRNA Fw | TTCCAGTGTGGCTGGTCATC | This study |
| 16sRNA Rw | TGCCTGATGGAGGGGGATAA | This study |
| modB Fw | TCGGTCTCACGTTTGCCTTT | This study |
| modB Rw | ATAATGCCCGGCAGGGTAAG | This study |
| ppdBFw | TTGCTCCGCGTAATGTCTCC | This study |
| ppdBRw | CCACTGTATTGGCGAAGGGT | This study |
| yggL Fw | CAGCCACTTACGCACAATCG | This study |
| yggL Rw | TTTCGGTGGCATGGCGTTTT | This study |
| ydiH Fw | TCAGTTCGGTTGCAGAGAGG | This study |
| ydiH Rw | CACGTTTACACATAAGCGAGGA | This study |
| SEN 1303 Fw | GTGGGGTCCGATTTCGTTCT | This study |
| SEN 1303 Rw | CCGCGAGGTAGCTGGAATAG | This study |
| scsAFw | GCGAAACAACAACGGATGGG | This study |
| scsaARw | ACTTGGCGCTTAATTCGCAG | This study |
| SEN1117Fw | AAACGATGCGTGGCATTCAG | This study |
| SEN1117Rw | AACGACCGTCACGGGTAATC | This study |
| ydeJFw | CCGTCTGACCACTGCAGAAT | This study |
| ydeJRw | GCCATTTCAGTAACGACCGC | This study |
| flgGFw | GGTTCTCGCCAATGCTTTCC | This study |
| flgGRw | CAATCACCATTCCGGCCAAC | This study |
| stfAFw | ATTGTCATCGGCAACCACCT | This study |
| stfARw | CGTGAAGTTTACGGTGCTGC | This study |
| stfFFw | AGCGGTTCCGTGAGTGATTT | This study |
| stfFRw | ATCGAAGCCGCCTTCTTTCA | This study |
| hdeBFw | TTCTCCTGTGCCAGCAACTC | This study |
| hdeBRw | CCAGCCGCAAATAGCCAATC | This study |
| SEN 1418 Fw | AGTAATCTGGCGAGGCGAAT | This study |
| SEN 1418 Rw | CAATGGTCACCGACCCAGAA | This study |
| SEN4193Fw | TCTTTGTTGGCCTGCTCTCC | This study |
| SEN4193Rw | GCGAACCTTCGATAATGCGG | This study |
| eutLFw | CGCCCAGCATAATCAACACC | This study |
| eutLRw | CGTAGCCTCGGACTCATCAC | This study |
| SEN1941Fw | GGTCAACAGTTTTCGCCTCAC | This study |
| SEN1941Rw | GAAGCGAGGGCTGGAAACAA | This study |
| SEN1963Fw | ACGGCATCACAGCAAGGTTA | This study |
| SEN1963Rw | TAGCCACACTAAGTCGGTGC | This study |
| yedOFw | ACCACCGCGGAAAATTACCT | This study |
| yedORw | CCGACCGGAATCACATAGGG | This study |
| yciGFw | TATCGGATTTACGTCCGCCG | This study |
| yciGRw | CCGAACATCGTGGTGGTTCA | This study |
| ymdFFw | CAAACCATCGTGGCGGTTC | This study |
| ymdFRw | GGTTACTGCTTTTGCCCCCT | This study |
| rpoSFw | GAGACGAAGCATACGGCTGA | This study |
| rpoSRw | CGATTCGCTTGCCGATTCAC | This study |

KO: Knockout ; Fw: Forward primer; Rw: Reverse primer ; Km/Kt: Internal reverse primer to Kanamycin Cassette ; Confo: Confirmatory forward primer for Knockout confirmation

**REFERENCES FOR SUPPLEMENTARY TABLE**

1. Ray S, Das S, Panda PK, Suar M. 2018. Identification of a new alanine racemase in *Salmonella* Enteritidis and its contribution to pathogenesis. Gut Pathog 10:1–17.

2. Das S, Ray S, Ryan D, Sahu B, Suar M. 2018. Identification of a novel gene in ROD9 island of *Salmonella* Enteritidis involved in the alteration of virulence-associated genes expression. Virulence 9:348–362.

**SUPPLEMENTARY FIGURES**

**
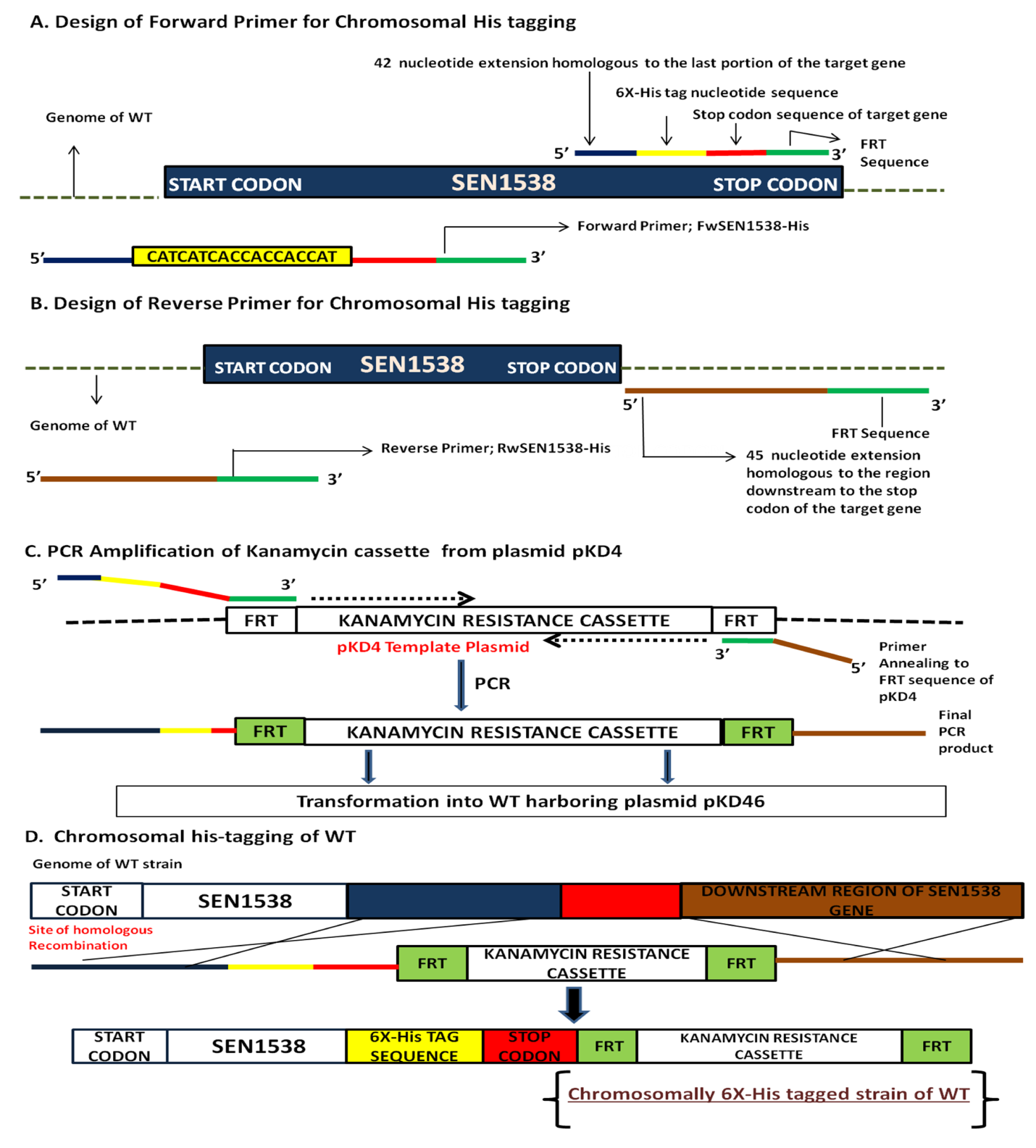
**

**Figure. S1. Chromosomal 6X-His Tagging of *SEN1538* gene.** *SEN1538* was tagged with 6X-His tag at its C-terminal end through a modified protocol of λ-red recombinase mutagenesis. (A) The forward primer comprised of a 5’ 42 nucleotide extension homologous to the last portion of *SEN1538* gene (Blue region of the forward primer), 6X-His tag nucleotide sequence (yellow region of the primer), stop codon sequence of *SEN1538* (red region of the primer) and a FRT sequence at 3’ end (green region of the primer).(B) The reverse primer comprised of a 5’ 45 nucleotide extension homologous to the downstream region of the stop codon of *SEN1538* gene (Brown region of the reverse primer) and a FRT sequence at its 3’ end ( green region of the primer). (C) Plasmid pKD4 harboring a kanamycin resistance cassette was used as the template for PCR. The FRT sequence of forward (green) and reverse primer (green) annealed to the FRT sequence in pKD4 to amplify the kanamycin cassette of size 1500 bp. The resulted amplicon had a 5’ overhang (blue sequence of the primer + yellow sequence of the primer + red sequence of the primer) and a 3’ overhang (brown). The PCR product was electroporated into WT harbouring helper plasmid pKD46. (D) The homologous regions of the PCR product and target genome served as the sites for recombination. The recombination resulted in substitution of kanamycin resistance cassette along with its 5’ end overhang sequence [6X-His tag sequence (Yellow) and the stop codon of the target gene (Red)] to the C- terminal end of *SEN1538* gene generating 6X-His tagged strain of WT (WT-SEN1538His).


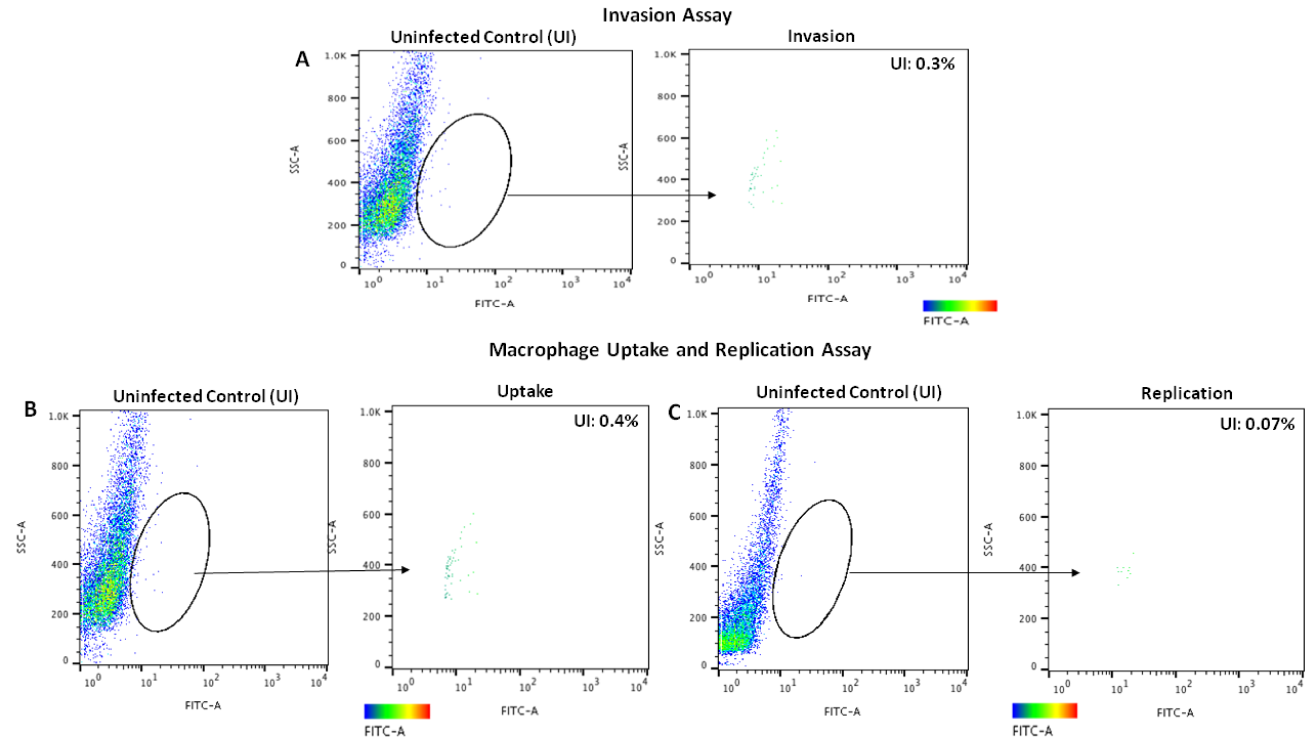


**
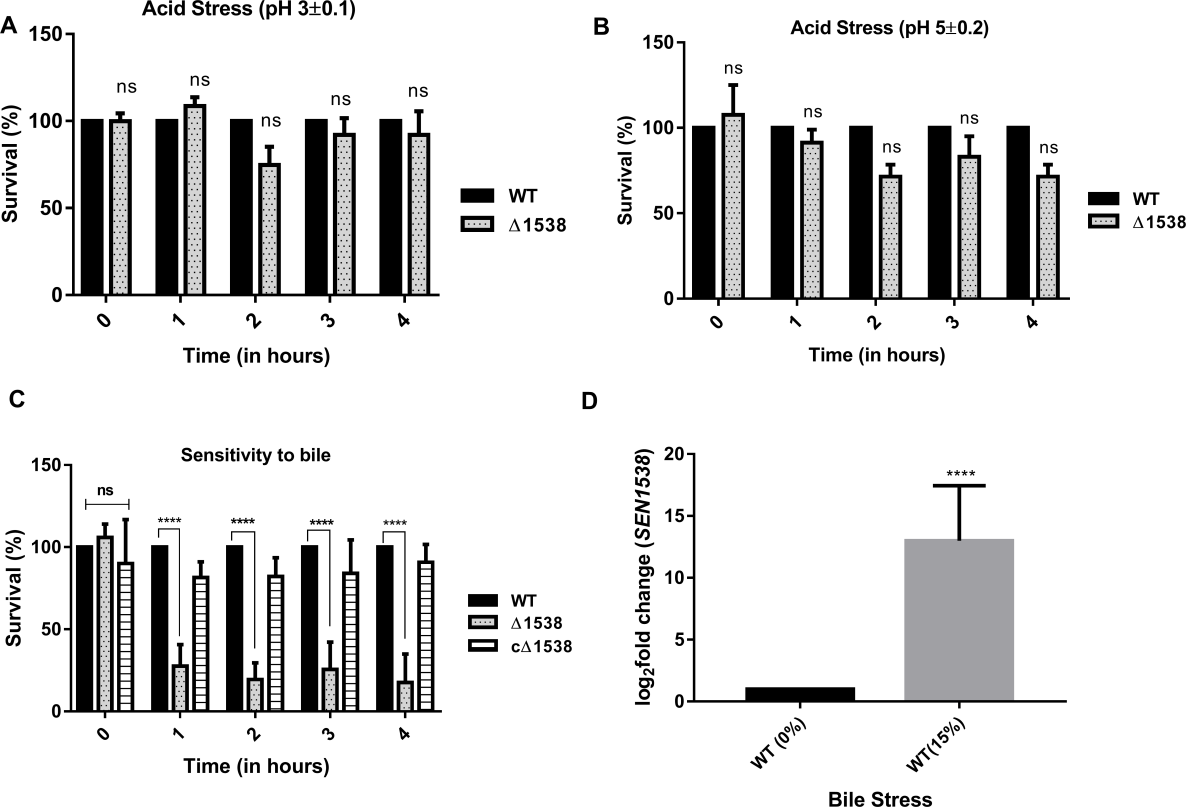
Figure. S2. Uninfected and non-flurorescent controls for flow cytometer experiments to validate Δ1538 invasion assay, macrophage uptake and replication assay.** Data were acquired using BD FACScanto™ II cytometer (Becton–Dickinson, Erembodegem, Belgium) and analyzed by using Flowjo v. 10.4.2.

**Figure. S3. SEN1538 promotes resistance to stressors.** Log phase culture of WT, Δ1538 and complemented strain cΔ1538 were grown in minimal medium and was challenged with (A) pH 3 (±0.1) (B) pH 5 (±0.2) and (C) Bile stress. (D)Expression of *SEN1538* in WT after exposure to bile stress through qRT-PCR. The bacterial counts were enumerated at indicated time points. Survivals (%) were compared to WT value normalized to 100%. *16srRNA* gene was taken as housekeeping gene in qRT-PCR experiments. Error bars indicate the mean±SD for three independent experiments. Statistical significance: ****, P < 0.0001; ns, not significant, P ≥ 0.05; Two-way ANOVA; Student’s t-test (Figure S2D).

**
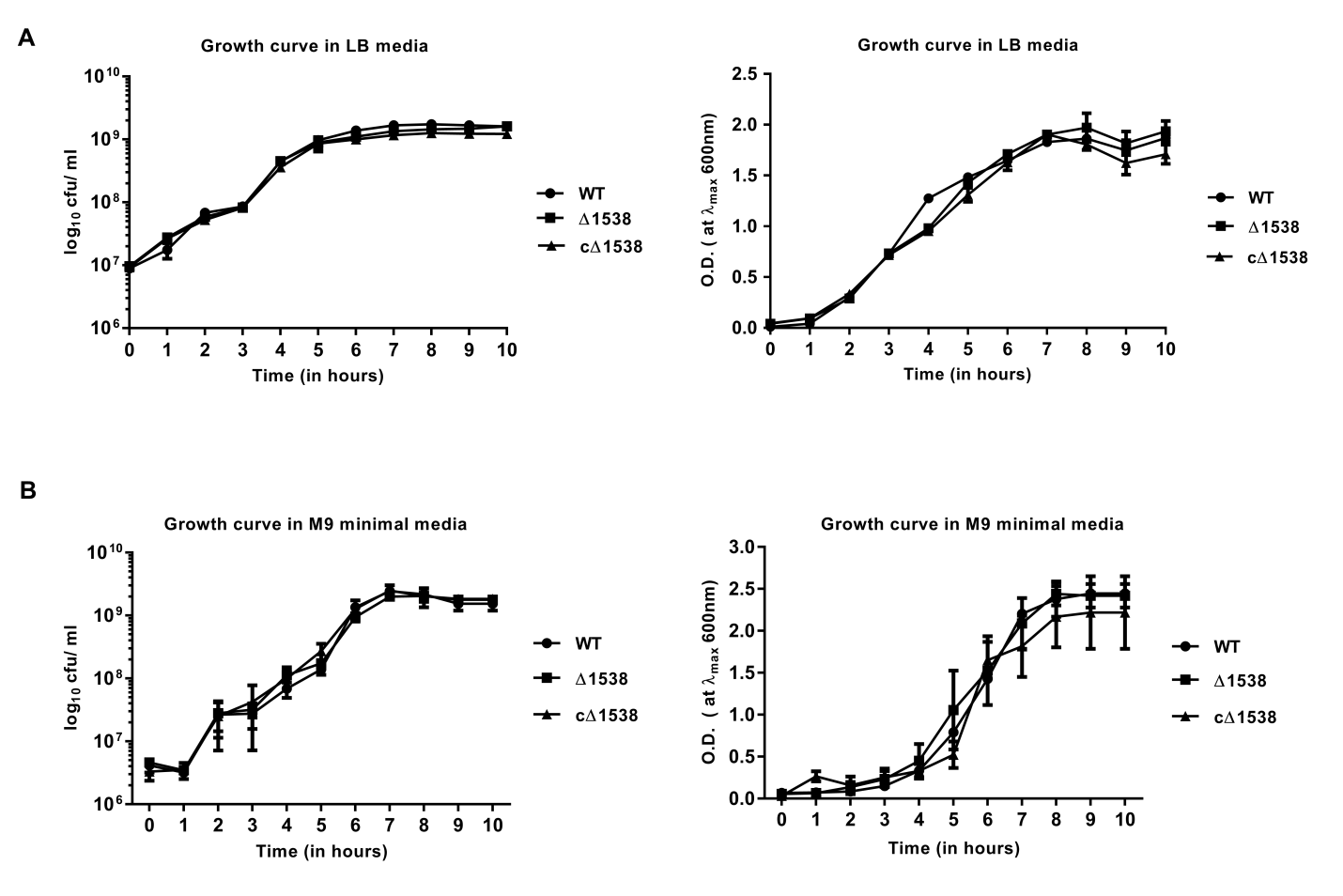
**

**Figure. S4. Growth curve analysis of WT, Δ1538 and cΔ1538.** (A) Growth curve analysis was of WT, Δ1538 and complemented strain cΔ1538 cultured in LB medium at 37 ˚C at 150 rpm upto 10 hours Growth analysis was determined in terms of log cfu / mL and O.D._600_ across indicated time points. (B) Growth curve analysis was of WT, Δ1538 and complemented strain cΔ1538 cultured in M9 minimal medium at 37˚C at 150 rpm upto 10 hours. Growth analyses were determined in terms of log cfu/mL and O.D._600_ across indicated time points. Results were representative of three independent experiments and data were represented as mean ± SD.


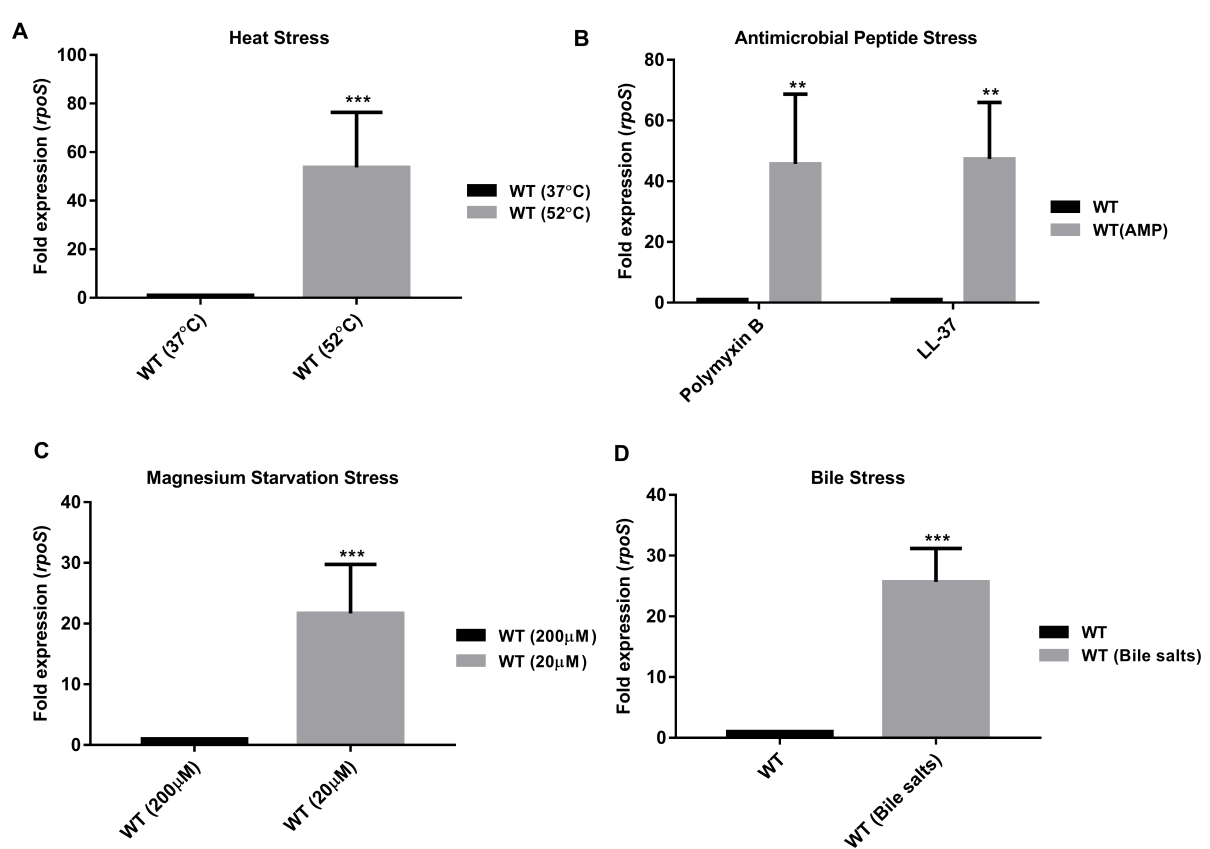


**Figure. S5. RpoS is highly expressed during stress.** Expression of *rpoS* was assessed in log phase culture of WT through qRT-PCR analysis during (A) Heat stress (B) AMP stress (C) Mg^2+^ starvation stress (D) Bile stress. *16srRNA* gene was taken as housekeeping gene. Results were representative of three independent experiments and data were represented as fold expression. Statistical significance: **, P < 0.01; ***P < 0.001 (Student’s t-test).


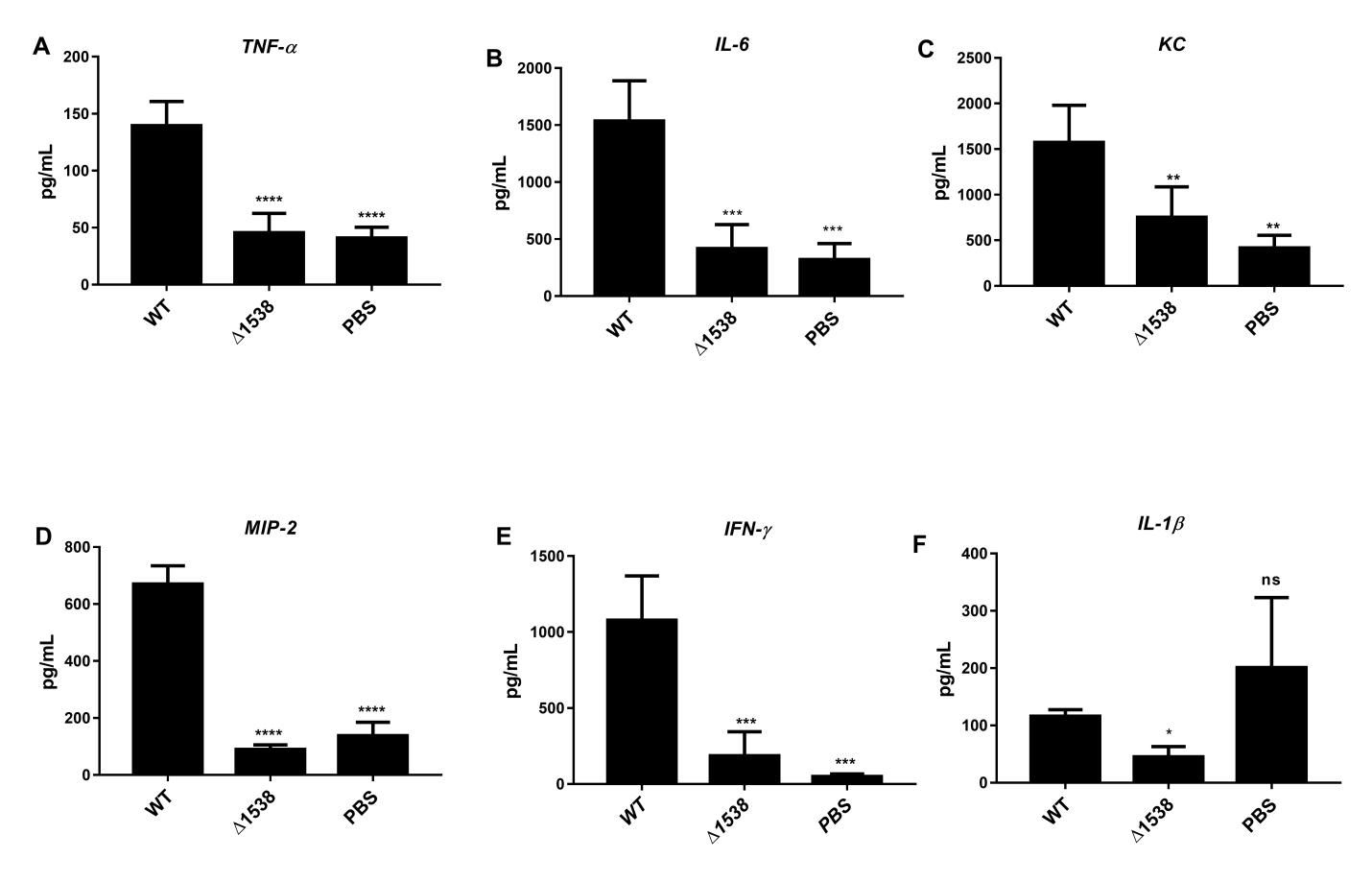


**Figure. S6. Motility assay.** 1.5µl of overnight and normalized cultures of (A) WT (B) Δ1538 (C) ΔyciG (D) SB300:ΔflgD (E) ΔymdF and (F) SB300: ΔyciG were spotted into (0.3% w/v) LB soft agar and grown at 37 °C. SB300:ΔflgD and SB300: ΔyciG were the experimental controls for the experiment. The diameter of motile cell growth for all strains were monitored after 5 hours and compared to WT. (G) Representative bar graph for the motility assay of strains. Each bar signified average ± SD of the motile cell growth of strains (in cm). The experiments were repeated in triplicates. Statistical significance: ****, P < 0.0001; ns, not significant , P ≥ 0.05 (one-way ANOVA).

**Figure. S7. Deletion of *SEN1538* reduces inflammation *in vivo*.** A serum cytokine assay was performed using the blood serum of mice. Serum from the infected mice groups (n=5) were collected at 72 hours p.i. and cytokine ELISA was conducted using a MILLIPLEX MAP Mouse Cytokine/Chemokine Magnetic Bead Panel - Premixed 32 Plex Immunology Multiplex Assay. Statistical significance: *, P < 0.05; **, P < 0.01; ***P < 0.001; ****, P < 0.0001; ns, not significant , P ≥ 0.05 (one-way ANOVA).

**
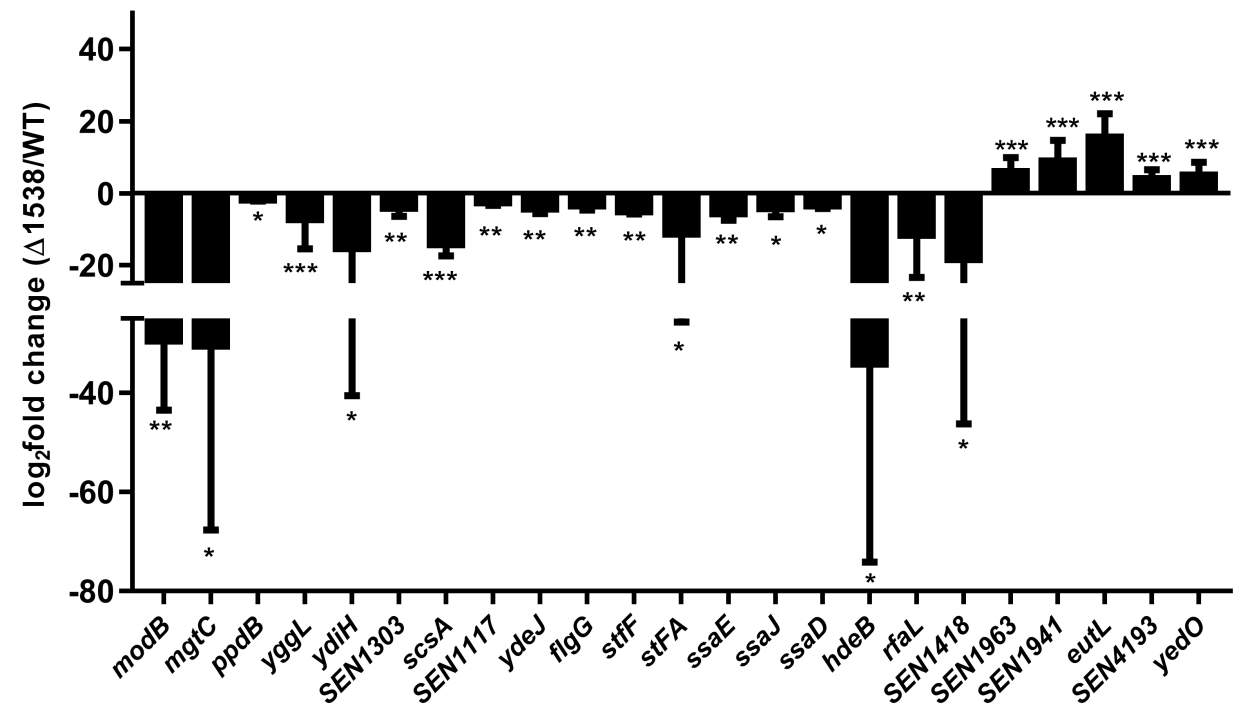
**

**Figure. S8. Validation of differentially expressed genes identified from RNA-seq through qRT-PCR analysis.** Twenty three differentially expressed (18 down-regulated; 5 up-regulated) genes were selected for qRT-PCR analysis and were compared to RNA-seq results. Data were represented as Log_2_ fold change difference in gene expression in Δ1538 compared to WT. Results were representative of three independent experiments. Statistical significance: *, P < 0.05; **, P < 0.01; ***P < 0.001(Student’s t-test).
